# Explorative characterization and taxonomy‐aligned comparison of alterations in lipids and other biomolecules in Antarctic bacteria grown at different temperatures

**DOI:** 10.1111/1758-2229.13232

**Published:** 2024-02-03

**Authors:** Volha Akulava, Margarita Smirnova, Dana Byrtusova, Boris Zimmermann, Dag Ekeberg, Achim Kohler, Uladzislau Blazhko, Uladzislau Miamin, Leonid Valentovich, Volha Shapaval

**Affiliations:** ^1^ Faculty of Science and Technology Norwegian University of Life Sciences Ås Norway; ^2^ Faculty of Chemistry, Biotechnology and Food Science Norwegian University of Life Sciences Ås Norway; ^3^ Faculty of Biology Belarussian State University Minsk Belarus; ^4^ Institute of Microbiology National Academy of Sciences of Belarus Minsk Belarus

## Abstract

Temperature significantly impacts bacterial physiology, metabolism and cell chemistry. In this study, we analysed lipids and the total cellular biochemical profile of 74 fast‐growing Antarctic bacteria grown at different temperatures. Fatty acid diversity and temperature‐induced alterations aligned with bacterial classification—Gram‐groups, phylum, genus and species. Total lipid content, varied from 4% to 19% of cell dry weight, was genus‐ and species‐specific. Most bacteria increased lipid content at lower temperatures. The effect of temperature on the profile was complex and more species‐specific, while some common for all bacteria responses were recorded. Gram‐negative bacteria adjusted unsaturation and acyl chain length. Gram‐positive bacteria adjusted methyl branching (anteiso‐/iso‐), chain length and unsaturation. Fourier transform infrared spectroscopy analysis revealed Gram‐, genus‐ and species‐specific changes in the total cellular biochemical profile triggered by temperature fluctuations. The most significant temperature‐related alterations detected on all taxonomy levels were recorded for mixed region 1500–900 cm^−1^, specifically the band at 1083 cm^−1^ related to phosphodiester groups mainly from phospholipids (for Gram‐negative bacteria) and teichoic/lipoteichoic acids (for Gram‐positive bacteria). Some changes in protein region were detected for a few genera, while the lipid region remained relatively stable despite the temperature fluctuations.

## Graphical abstract

Temperature influences polar bacteria's biochemistry. In this study, 74 Antarctic bacteria grown at different temperatures were analysed. Fatty acid diversity and temperature‐induced alterations aligned with bacterial classification—Gram‐groups, phylum, genus and species. The complex species‐specific impact of temperature was observed, as well as some responses common for all bacteria were recorded. Fourier transform infrared spectroscopy analysis revealed taxonomy‐specific changes triggered by temperature fluctuations, particularly related to phosphodiester groups mainly from phospholipids and teichoic/lipoteichoic acids with minor protein variations and stable lipid regions despite temperature fluctuations.

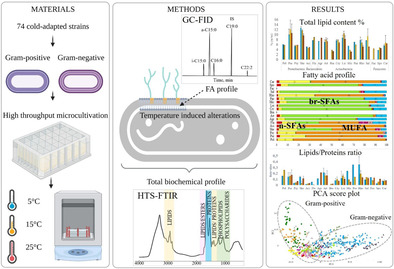

## INTRODUCTION

Psychrotrophic and psychrophilic bacteria have garnered attention due to biomolecules they can produce which have application potential in biotechnology and medicine. Their ability to survive and thrive in frigid environments, such as polar regions, often relies on alterations in cellular lipids and the production of specific compounds, such as antifreeze proteins (De Maayer et al., 2014), cold‐active enzymes, cryoprotection‐targeted exopolysaccharides, compatible solutes (Collins & Margesin, 2019), storage compounds and pigments (Sajjad et al., 2020). Some bacterial fatty acids (FAs) and monoglycerides are promising antibacterial agents due to that they destabilize bacterial cell membranes, resulting in a variety of direct and indirect inhibitory effects (Desbois & Smith, 2010; Yoon et al., 2018). Finally, the accumulation of storage compounds such as acyl glycerides and polyhydroxyalkanoates (PHAs) has been reported as an adaptation to low temperatures and nutrient‐poor conditions (Goh & Tan, 2012; Tribelli & López, 2018).

Lipids are one of the main temperature‐sensitive biomolecules in bacterial cells which account for approximately 10%–15% (w/w) of cell dry weight (Naumann, 2000). They are localized mainly in the form of phospholipids in the cell membrane or can be accumulated in the form of acyl glycerides and/or free FAs in lipid droplets. Lipids play multiple roles in bacterial cells such as membrane flexibility, selective permeability and establishment of the environment for many enzyme and protein transport (Chattopadhyay & Jagannadham, 2001). The fatty acid profile is considered a chemotaxonomy biomarker used for bacterial identification on genus and species level (Sasser, 1990).

For cold‐adapted bacteria, temperature‐associated alterations in the amount of lipids, the ratio between different types of lipids, and fatty acid profiles have been reported previously (Hassan et al., 2020). Modification of fatty acid composition and ratios of different FAs impact the fluidity, flexibility, and permeability of cell membranes resulting in the elevated survival rate at low and high temperatures. Thus, increased production of saturated fatty acids (SFAs) and cyclopropane fatty acids (cyclic‐FA) can increase rigidity and lower permeability of membrane bilayer, while high presence of cis‐unsaturated fatty acids (cis‐UFAs) can lead to a higher permeability of membrane (Mező et al., 2022). Changes in branched‐chain fatty acids can affect membrane fluidity, where an increase in anteiso‐fatty acids (anteiso‐FA) can result in a more fluid membrane structure than for iso‐fatty acids (iso‐FA) (Mező et al., 2022). The ratio between long‐ and short‐chain fatty acids can also regulate membrane fluidity under unfavourable temperature conditions the same as the change of saturation. Moreover, temperature fluctuations can lead to the conversion of cis‐UFAs into their corresponding trans configurations, leading to a quick rigidification of the cell membrane (Mező et al., 2022).

Proteins and carbohydrates are among the primary biomolecules found in bacterial cells that exhibit temperature sensitivity. Proteins are the main components of bacterial cells accounting for approximately 40%–60% (w/w) of cell dry weight (Naumann, 2000). Temperature fluctuations can impact protein structures and activity. Antarctic bacteria thrive in lower temperatures, producing cold‐adapted proteins that maintain stability, flexibility, and enhanced catalytic activity. These bacteria also produce antifreeze proteins that bind to ice surfaces, preventing ice crystal formation and enabling survival in freezing conditions (De Maayer et al., 2014). Polysaccharides accounting for approximately 10%–20% (w/w) of cell dry weight (Naumann, 2000) can significantly be affected by temperature and alter production, composition, and structure. Lower temperatures commonly lead to increased production of exopolysaccharides and induce modifications that contribute to bacterial survival and adaptation to extreme cold conditions (De Maayer et al., 2014; Tribelli & López, 2018).

The primary objective of this study was to perform explorative characterization and taxonomy‐aligned comparison of the temperature‐induced alterations of the main biomolecules such as lipids, proteins, and polysaccharides in Antarctic cold‐adapted bacteria newly isolated from green snow and temporary meltwater ponds belonging to four phyla and eighteen genera. Total lipid content and fatty acid profiles were analysed by gas chromatography (GC), while Fourier transforms infrared (FTIR) spectroscopy was used to evaluate changes in the total cellular biochemical profile. FTIR spectroscopy was chosen due to its advantages and wide application in microbiology and biotechnology for the overall biochemical characterization of microbial cells and their metabolites (Carnovale et al., 2021; Forfang et al., 2017; Kohler et al., 2015; Kosa, Shapaval, et al., 2017; Olsen et al., 2023; Shapaval et al., 2017, 2023). One of the main advantages of FTIR analysis is that it can be performed on non‐destructed or little‐processed microbial biomass and combined with automated sample preparation to increase throughput (Li et al., 2016; Xiong et al., 2019).

## EXPERIMENTAL PROCEDURES

### Bacterial strains

Seventy‐four fast‐growing Antarctic bacteria from the Belarussian Collection of Non‐pathogenic Microorganisms (Institute of Microbiology of the National Academy of Science of Belarus) were used in the study. These are Gram‐positive and Gram‐negative, psychrotrophic and psychrophilic bacteria belongs to four phyla: *Proteobacteria*, *Actinobacteria*, *Firmicutes* and *Bacteroidetes* and represented by 18 genera: *Arthrobacter*, *Cryobacterium*, *Leifsonia*, *Salinibacterium*, *Paeniglutamicibacter*, *Rhodococcus*, *Polaromonas*, *Pseudomonas*, *Psychrobacter*, *Shewanella*, *Acinetobacter*, *Sporosarcina*, *Facklamia*, *Carnobacterium*, *Brachybacterium*, *Micrococcus*, *Agrococcus* and *Flavobacterium* (Table 1 and Table S1 in the Supporting Information). Identification and physiological characterization of these bacteria have been reported previously (Akulava et al., 2022, 2024; Smirnova et al., 2021, 2022, 2023).

**TABLE 1:** List of bacterial strains used in the study.

Genus	Strain name and collection No	*T*°C		
		5	15	25
Gram‐negative				
Proteobacteria				
Pol	*Polaromonas* sp. BIM B–1676 ^GS^**			X
Pse	*Pseudomonas extremaustralis* BIM B–1672 ^GS^			
	*Pseudomonas fluorescens* BIM B–1668 ^GS^			
	*Pseudomonas leptonychotis* BIM B–1559^MP^			
	*Pseudomonas leptonychotis* BIM B–1568^MP^			
	*Pseudomonas leptonychotis* BIM B–1566^MP^			
	*Pseudomonas lundensis* BIM B–1554^MP^			
	*Pseudomonas lundensis* BIM B–1555^MP^			
	*Pseudomonas lundensis* BIM B–1556^MP^			
	*Pseudomonas peli* BIM B–1560^MP^			
	*Pseudomonas peli* BIM B–1569^MP^			
	*Pseudomonas peli* BIM B–1546 ^MP^			
	*Pseudomonas peli* BIM B–1552^MP^			
	*Pseudomonas peli* BIM B–1542^MP^			
	*Pseudomonas peli* BIM B–1548^MP^			
	*Pseudomonas* sp. BIM B–1635^GS^			
	*Pseudomonas* sp. BIM B–1667^GS^			
	*Pseudomonas* sp. BIM B–1673^GS^			
	*Pseudomonas* sp. BIM B–1674^GS^			
Psy	*Psychrobacter glacinicola* BIM B–1629^GS^**			X
	*Psychrobacter urativorans* BIM B–1655 ^GS^**			X
	*Psychrobacter urativorans* BIM B–1662^GS^			
She	*Shewanella baltica* BIM B–1565^MP^			
	*Shewanella baltica* BIM B–1557^MP^			
	*Shewanella baltica* BIM B–1561^MP^			
	*Shewanella baltica* BIM B–1563^MP^			
Aci	*Acinetobacter lwoffii* BIM B–1558^MP^			
Bacteroidetes				
Fla	*Flavobacterium degerlachei* BIM B–1562^MP^**			X
Gram‐positive				
Actinobacteria				
Agr	*Agrococcus citreus* BIM B–1547^MP^		/	
Art	*Arthrobacter agilis* BIM B–1543 ^MP^			
	*Arthrobacter cryoconiti* BIM B–1627^GS^			
	*Arthrobacter oryzae* BIM B–1663^GS^			
	*Arthrobacter* sp. BIM B–1624^GS^			
	*Arthrobacter* sp. BIM B–1625 ^GS^			
	*Arthrobacter* sp. BIM B–1626^GS^			
	*Arthrobacter* sp. BIM B–1628^GS^			
	*Arthrobacter* sp. BIM B–1664^GS^			
	*Arthrobacter* sp. BIM B–1666^GS^**			X
	*Arthrobacter* sp. BIM B–1656^GS^			
	*Arthrobacter* sp. BIM B–1549^MP^			
Bra	*Brachybacterium paraconglomeratum* BIM B–1571 ^MP^	X		
Cry	*Cryobacterium arcticum* BIM B–1619 ^GS^			
	*Cryobacterium soli* BIM B–1620 ^GS^			
	*Cryobacterium soli* BIM B–1658^GS^			
	*Cryobacterium soli* BIM B–1659^GS^			
	*Cryobacterium soli* BIM B–1677^GS^			
	*Cryobacterium soli* BIM B–1675^GS^			
Lei	*Leifsonia antarctica* BIM B–1631^GS^	X		
	*Leifsonia antarctica* BIM B–1632^GS^			
	*Leifsonia antarctica* BIM B–1637 ^GS^			
	*Leifsonia antarctica* BIM B–1638 ^GS^			
	*Leifsonia antarctica* BIM B–1639 ^GS^			
	*Leifsonia antarctica* BIM B–1669 ^GS^			
	*Leifsonia antarctica*. BIM B–1671^GS^			
	*Leifsonia kafniensis* BIM B–1633^GS^		/	
	*Leifsonia rubra* BIM B–1622 ^GS^			
	*Leifsonia rubra* BIM B–1623 ^GS^**	X		X
	*Leifsonia rubra* BIM B–1634 ^GS^	X		/
	*Leifsonia rubra* BIM B–1567^MP^			
Mic	*Micrococcus luteus* BIM B–1545^MP^			
Pae	*Paeniglutamicibacter antarcticus* BIM B–1657^GS^**			X
Rho	*Rhodococcus erythropolis* BIM B–1660^GS^			
	*Rhodococcus erythropolis* BIM B–1661^GS^			
	*Rhodococcus yunnanensis* BIM B–1621^GS^**			X
	*Rhodococcus yunnanensis* BIM B–1670^GS^			
Sal	*Salinibacterium* sp. BIM B–1630 ^GS^**	X		X
	*Salinibacterium* sp. BIM B–1636^GS^	X	X	/
	*Salinibacterium* sp. BIM B–1654^GS^**	X		X
	*Salinibacterium* sp. BIM B–1665^GS^**			X
Firmicutes				
Fac	*Facklamia tabacinasalis* BIM B–1577^MP^	X		X
Spo	*Sporosarcina* sp. BIM B–1539 ^MP^			
Car	*Carnobacterium funditum* BIM B–1541^MP^**			X
	*Carnobacterium iners* BIM B–1544^MP^**			X
	*Carnobacterium inhibens* BIM B–1540^MP^			

*Note*: X—no growth; /—not enough biomass for analysis; **—psychrophilic bacteria based on growth on BHI broth media; ^GS^—green snow bacteria. ^MP^—temporary meltwater ponds bacteria.

### Microscopy evaluation of gram‐stained bacteria

For Gram staining and microscopy evaluation, the isolated bacteria were cultivated on brain heart infusion (BHI)‐agar at 18°C for 1–4 days, depending on the isolate, until the single colony appeared. Gram staining was done following the protocol of the three‐step Gram stain procedure kit (Merck KGaA, Germany). The morphology of Gram‐stained cells was studied by direct examination with the light microscope Leica DM4 B (Leica Microsystems, Germany) under a 100× immersion lens.

### Cultivation of Antarctic bacteria

Bacteria were recovered from cryo‐preserved cultures by culturing on BHI agar (Sigma Aldrich, USA) plates for 7 days at 18°C. A single colony of each strain was transferred into 7 mL BHI broth (Sigma‐Aldrich, India) in the Duetz microtiter plate system (Duetz‐MTPS, Enzyscreen, the Netherlands) consisting of 24‐square extra high polypropylene deep well microtiter plates (MTPs) with low‐evaporation sandwich covers and extra high cover clamps (Duetz et al., 2000; Dzurendova et al., 2020a; Dzurendova et al., 2020b; Kosa, Kohler, et al., 2017). To obtain enough amount of biomass for analysis, each strain was inoculated into four wells of an MTP. Inoculated MTPs were mounted on the shaking platform of the MAXQ 4000 incubator (Thermo Fisher Scientific, Waltham, MA, USA), incubated at 5°C, 15°C and 25°C, and 400 rpm agitation speed (1.9 cm circular orbit) for 7 days. One well in each plate was filled with a sterile medium for cross‐contamination control. All cultivations were done in two independently performed biological replicates.

### Preparation of bacterial biomass for FTIR measurements

Bacterial biomass was separated from the growth medium by centrifugation (Heraeus Multifuge X1R, Thermo Scientific, Waltham, MA, USA) at 2490*g* 4°C for 10 min and washed with distilled water three times. Further, at the last washing step, 100–500 μL of distilled water was added to the cell pellet and re‐suspended. About 10 μL of the homogenized bacterial suspension was pipetted onto the IR‐light‐transparent silicon 384‐well silica microplates (Bruker Optics GmbH, Ettlingen, Germany) in three technical replicates, and dried at room temperature for at least 2 h before the analysis (Smirnova et al., 2021; Smirnova et al., 2022). The remaining bacterial biomass was freeze‐dried (Labconco, USA) for 72 h until constant weight, and stored at −20°C. Freeze‐dried biomass was used for lipids extraction.

### Lipid extraction from bacterial biomass

Lipid extraction was done by using the previously described method (El Razak et al., 2014), with some modifications. Briefly, 20 mg of freeze‐dried bacterial biomass was mixed with 2 mL of 8% methanolic HCl (Ichihara & Fukubayashi, 2010) in reaction glass tubes. Further, 50 μL of 19:0 1,2‐dinonadecanoyl‐sn‐glycero‐3‐phosphocholine (PC) internal standard solution in chloroform (10 mg/mL) (Avanti, USA) was added to each sample (Quideau et al., 2016). Samples were heated at 70°C for 2 h and cooled down at room temperature for 30 min. About 1 mL of distilled water was added to the samples and vortexed. Phase separation was performed two times: 2 mL of hexane was added to the samples, vortexed for 1 min and centrifuged at 1968*g* for 5 min. The upper hexane phase was transferred into clean glass tubes and evaporated under nitrogen at 30°C (SBH130D/3N2 evaporator, ColePalmer™ Stuart™). Fatty acid methyl esters (FAMEs) were transferred into a GC vial by washing the glass tube with 1500 μL hexane containing 0.01% butylated hydroxytoluene (BHT, Sigma‐Aldrich, USA), followed by 5 s vortex mixing at low speed.

### Gas chromatography analysis of total lipid content and fatty acid profile

Lipid contents and fatty acid profiles were analysed using GC 820A System (Agilent Technologies, Santa Clara, CA, USA) equipped with Agilent J&W 121‐2323 DB‐23 column, 20 m × 180 μm × 0.20 μm and a flame ionization detector (FID). Helium as a carrier gas was used. Setup for sample analysis was used as described previously (Langseter et al., 2021). For the identification and quantification of fatty acids, the C4–C24 FAME mixture (Supelco, St. Louis, MO, USA) and bacterial acid methyl esters (BAMEs) CP mixture (Matreya LLC, High Tech Road, State College, PA 16803, USA) were used as an external standard, in addition to C19:0 PC internal standard. Gas chromatography–mass spectrometry (GC–MS) analysis of the fatty acid profile was used to identify fatty acids that were not present in the external standards used for GC–FID, and this was done as described previously (Kosa, Zimmermann, et al., 2018).

### FTIR spectroscopy analysis

FTIR transmittance spectra were measured using a high‐throughput screening extension unit (HTS‐XT) coupled to the Vertex 70 FTIR spectrometer (both Bruker Optik, Germany). The FTIR system was equipped with a global mid‐IR source and a deuterated L‐alanine‐doped triglycine sulfate (DLaTGS) detector. The HTS‐FTIR spectra were recorded with a total of 64 scans, using Blackman‐Harris 3‐Term apodization, spectral resolution of 6 cm^−1^, and digital spacing of 1.928 cm^−1^, over the range of 4000–400 cm^−1^, and an aperture of 6 mm. The ratio of a sample spectrum to a spectrum of the empty IR transparent microplate was used to calculate the final spectrum. Background spectra of the Si microplate were collected before each sample measurement to account for variations in water vapour and CO_2_. Generated transmittance spectra were exported for further analysis. Each biomass sample was analysed in three technical replicates. For data acquisition and instrument control, the OPUS software (Bruker Optik GmbH, Germany) was used.

### Preprocessing and data analysis

#### GC data

The weight of individual fatty acids (FAs) was calculated based on peak areas, relative response factors (RRFs), and C19:0 internal standard. The total lipid content of bacterial biomass was estimated as a sum of FAMEs (the weight of C19:0 was subtracted) divided by the weight of dry biomass. The total lipid content of the biomass was calculated in a percentage (%) by summing up all detected FAs for the whole set of studied strains individually. Detected FAs were grouped according to their structural characteristics: PUFAs (summed polyunsaturated fatty acids), n‐SFAs (summed non‐branched saturated fatty acids), br‐SFAs (summed branched saturated fatty acids), n‐MUFAs (summed non‐branched monounsaturated fatty acids), hydroxy‐FAs (summed hydroxy fatty acids), cyclic‐FAs (summed cyclic fatty acids), summed cis‐FAs/trans‐FAs and iso‐FAs/anteiso‐FAs (Mező et al., 2022). Before principal component analysis (PCA), GC fatty acid profile data were normalized by using autoscaling with mean‐centring, followed by the division of each column (variable) by the standard deviation. PCA analysis was performed without any prior knowledge about the experimental structure to uncover structural relationships between the variables and identify potential clusters in the data.

#### FTIR data

For PCA analysis, HTS‐FTIR spectra of the bacterial biomass were preprocessed in the following way: (1) applying the Savitzky–Golay algorithm using a polynomial order of degree 2 and window size 11 (Savitzky & Golay, 1964), (2) cutting uninformative regions (4000–3100, 2800–1800 and 900–400 cm^−1^), (3) the extended multiplicative signal correction (EMSC) was applied to the second‐derivative spectra to separate informative signals from spectral artefacts and minimize variability due to the light scattering or sample thickness (Kohler et al., 2020; Tafintseva et al., 2020). For ratio analysis, FTIR‐HTS spectra were preprocessed in the following way: (1) applying the Savitzky–Golay algorithm using a polynomial order of degree 2 and window size 11 (Savitzky & Golay, 1964), selecting an informative region (1900–900 cm^−1^). The spectral data analysis involved categorizing the spectra into specific regions: lipids (3050–2800 cm^−1^), esters (1800–1700 cm^−1^), proteins (1700–1500 cm^−1^) and a mixed region (1500–900 cm^−1^).

After preprocessing, the infrared spectra were subjected to multivariate analysis using PCA. For the PCA, the whole spectral region was used. The scatter plot of scores was generated for the entire FTIR dataset, including biological and technical replicates, which was then projected onto a PCA plot. Univariate analysis of the infrared spectra was used to estimate the relative content of lipids, phosphorus‐containing compounds (i.e., phospholipids), and changes in protein structure, where the amide I peak at 1656 cm^−1^ related to the α‐helical structure of proteins was selected as a relatively stable reference band. An ester C=O stretching peak at 1742 cm^−1^ was used for the estimation of the relative lipid content (lipid to protein ratio, L/P, 1742 cm^−1^/1656 cm^−1^), while P‐O‐C symmetric stretching peak at 1083 cm^−1^ was used for the estimation of phosphorus‐containing compounds (phosphorus to protein ratio, P/P, 1083/1656 cm^−1^) (Garip et al., 2009; Maquelin et al., 2002; Naumann, 2000). Orange data mining toolbox version 3.31.1 (University of Ljubljana, Ljubljana, Slovenia) was used for the preprocessing and spectral analysis (Demšar et al., 2013; Toplak et al., 2017).

## RESULTS

The bacteria isolated from Antarctic meltwater ponds were Gram stained and cell morphologies were studied by microscopy. Gram staining showed that among 29 isolates from meltwater ponds, 18 isolates were Gram‐negative and 11 isolates were Gram‐positive. The microscopy images for the 12 isolates representing all genera are shown in Figure S1 in the Supporting Information. Microscopic examination of the Gram‐stained bacteria revealed a predominance of the cocci‐shaped cells such us for *Acinetobacter lwoffii* BIM B‐1558 and *Facklamia tabacinasalis* BIM B‐1577 or short bacilli‐shaped cells as for *Shewanella baltica* BIM B‐1563, *Pseudomonas peli* BIM B‐1560, *Sporosarcina* sp. BIM B‐1539, *Arthrobacter* sp. BIM B‐1549 and *Leifsonia rubra* BIM B ‐1567, while for *Flavobacterium degerlachei* BIM B‐1562 and *Carnobacterium funditum* BIM B ‐1541 peculiar cell morphology in the form of threads was more characteristic (Figure S1 in the Supporting Information). The result of Gram staining for the green snow bacteria was previously reported (Smirnova et al., 2021) and indicated that among 45 isolates, 33 isolates were Gram‐positive and 12 isolates were Gram‐negative. Thus, in total, in this study 45 were Gram‐positive and 30 isolates were Gram‐negative.

To perform explorative characterization of temperature‐triggered alterations of cellular biomolecules, bacteria were grown in a BHI nutrient‐rich broth medium. Notably, the growth performance of some bacteria under disparate temperature conditions in broth media differed from previous observations made on agar media. Among the studied bacteria, 55 showed good growth at all three temperatures used, while eight isolates did not exhibit growth at temperatures 5°C or/and 15°C. Additionally, 15 isolates were only able to grow at 25°C (Table 1). Proteobacteria demonstrated robust growth across a range of tested temperatures, except psychrophilic strains from genera *Polaromonas* and *Psychrobacter*, which were unable to grow at 25°C. In contrast, some Actinobacteria (*Salinibacterium* and *Paeniglutamicibacter*) and Firmicutes (*Carnobacterium*) exhibited greater temperature sensitivity. Psychrophilic bacteria were defined by their ability to thrive at a maximum growth temperature of 18°C and did not grow at 25°C based on the previous definition done by (Morita, 1975).

It can be seen that growth ability at different temperatures was more genus and species‐specific. For example, Actinobacteria from the genus *Salinibacterium* were not able to grow in liquid culture at 5°C and 25°C but grew well on agar media (Smirnova et al., 2022). Strains from the genus *Salinibacterium* and some species, such as *Leifsonia rubra* and *Facklamia tabacinasalis*, exhibited heightened susceptibility to both high (25°C) and low (5°C) temperatures, with better growth occurring exclusively at 15°C. Strains within genera *Polaromonas*, *Psychrobacter*, *Flavobacterium*, *Carnobacterium*, *Rhodococcus*, *Salinibacterium* and *Paeniglutamicibacter* were identified as psychrophiles (did not grow at 25°C), according to their growth in broth media. On the other hand, some psychrophilic strains within genera *Psychrobacter*, *Arthrobacter*, *Cryobacterium* and *Leifsonia* did not appear to be psychrophilic when grown in broth media and displayed an ability to withstand 25°C (Table 1).

### Changes in total lipid content

The BHI broth is a rich and complex medium that may contain some lipidic compounds that may affect the lipid profile of bacteria. To exclude this, we analysed the overall biochemical composition of BHI broth by FTIR spectroscopy and we did not observe any lipid‐related peaks on FTIR spectra, especially the peak at 1745 cm^−1^ related to C=O vibrations in lipids and used for estimating relative total lipid content, was not detected (Figure S9 in the Supporting Information).

**FIGURE 1:**
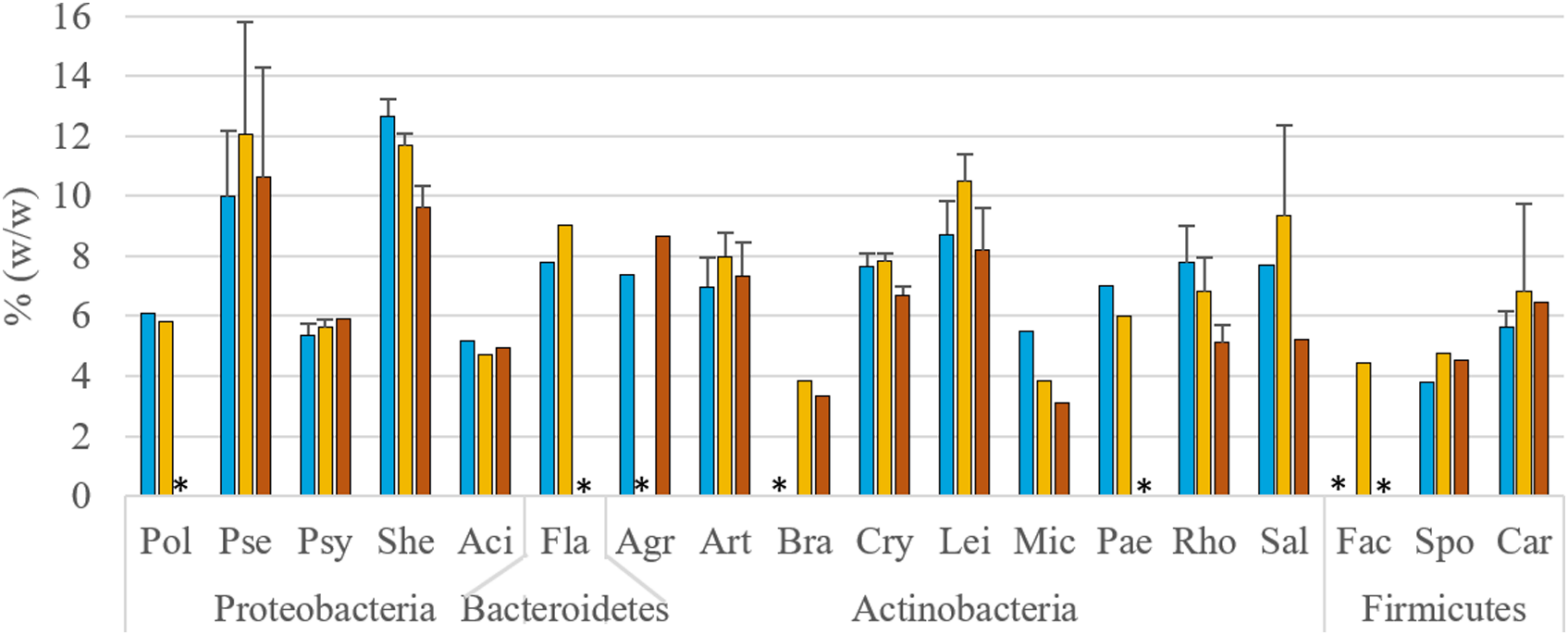
Total lipid content (%, w/w) of bacterial biomass of different genera grown at different temperatures (blue—5°C, yellow—15°C and orange—25°C), *—no growth or not enough biomass to perform the analysis. The standard deviation was calculated for genera that were represented by two or more strains; Genera: Pse—*Pseudomonas*, Psy—*Psychrobacter*, She—*Shewanella*, Aci—*Acinetobacter*, Art—*Arthrobacter*, Cry—*Cryobacterium*, Lei—*Leifsonia*, Mic—*Micrococcus*, Rho—*Rhodococcus*, Sal—*Salinibacterium*, Spo—*Sporosarcina*, Car—*Carnobacterium*.

Total lipid content for the two Gram groups differed, where Gram‐negative bacteria exhibited on average a higher total lipid content compared to Gram‐positive bacteria (Figure S14 in the Supporting Information). Further, Proteobacteria displayed the highest total lipid content compared to other phyla. However, the main variability in total lipid content was observed among genera within a single phylum and among species within a single genus. (Figure S2 in the Supporting Information). Bacterial isolates from genera *Pseudomonas*, *Shewanella*, *Leifsonia* and *Salinibacterium* showed relatively high total lipid content from 10%_w/w_ to 19%_w/w_ (% of cell dry weight) (Figure 1 and Figure S2 in the Supporting Information). The highest total lipid content was recorded for *Pseudomona*s isolates grown at 15°C, where *Pseudomonas peli* strains showed the highest values (Figure 1 and Figure S2 in the Supporting Information). Bacteria from genera *Polaromonas*, *Psychrobacter*, *Acinetobacter*, *Brachybacterium*, *Micrococcus*, *Facklamia* and *Sporosarcina* had relatively low total lipid content, below 6%_w/w_, and for all other bacteria, it was between 6%_w/w_ and 10%_w/w_ (Figure 1). It was observed that total lipid content was more genera‐specific except for the genus *Pseudomonas*, where it considerably varied from 6%_w/w_ to 19%_w/w_ between different species (Figure 1 and Figure S2 in the Supporting Information).

Average total lipid content was higher at 15°C compared to growth at 5°C/25°C, but big variations between the genera were observed (Figure S14 in the Supporting Information). The effect of temperature on the total lipid content was found to be genus and species‐specific, with no common effect observed at the phylum or Gram‐group level. Additionally, genus‐ and species‐specific changes can be seen, where bacterial strains of the same genus or species showed similar temperature‐induced changes. For example, total lipid content increased for all isolates *Shewanella*, *Micrococcus* and *Rhodococcus* when grown at 5°C/15°C, and for *Pseudomonas*, *Arthrobacter*, *Cryobacterium*, *Leifsonia*, *Salinibacteriu* and *Carnobacterium* at 15°C (Figure 1 and Figure S2 in the Supporting Information). Species‐specific temperature‐triggered changes were observed for *S. baltica*, *Pseudomonas lundensis*, *Leifsonia antarctica* and *Cryobacterium soli* (Figure S2 in the Supporting Information).

Psychrophilic Proteobacteria related to *Polaromonas*, and *Psychrobacter* genera exhibited the lowest total lipid content, as depicted in Figure S2 in the Supporting Information. Within the phylum Actinobacteria, the majority of psychrophilic strains showed total lipid content similar to psychrotrophic bacteria. In contrast, psychrophilic bacteria from phylum Firmicutes displayed lower total lipid content at 15°C. Our comparative analysis of psychrophiles and psychrotrophs revealed that for most of the tested strains alterations in the total lipid content were similar for psychrotrophic bacteria. Generally, little effect of temperature on the total lipid content was observed for the studied Antarctic psychrophilic strains, which is an indication of the remarkable stability of the total lipid content across the range of temperatures tested for these bacteria.

Interestingly, the lipid content of Proteobacteria from genera *Polaromonas*, *Psychrobacter* and *Acinetobacter* was consistent regardless of cultivation temperature, and only a few strains showed a slight increase at 25°C (Figure 1 and Figure S2 in the Supporting Information). *Pseudomonas* sp. strain BIM B‐1635 was unique, with a relatively high increase of total lipid production at high temperatures (16%_w/w_ at 25°C, compared to 10%_w/w_ at 5°C) (Figure S2 in the Supporting Information).

### Temperature effect on taxonomic diversity of fatty acid profile

**FIGURE 2:**
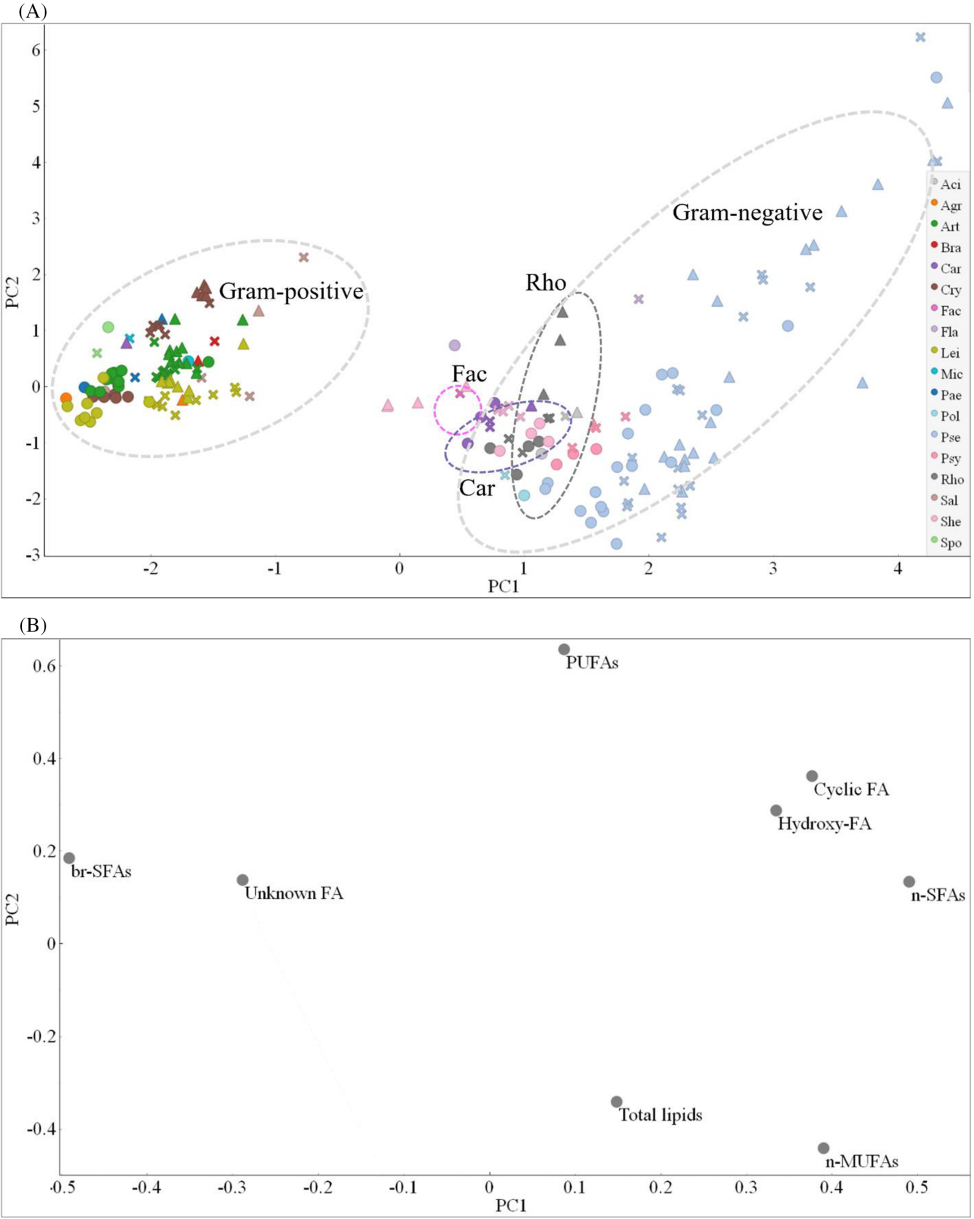
PCA of the GC fatty acid (FA) profile data for Antarctic bacteria grown at different temperatures. (A) Score plot of PC1 and PC2, colours represent genera, ‘●’—5°C, ‘✖’—15°C and ‘▲’—25°C, different colours represent genera (Pol—*Polaromonas*, Pse—*Pseudomonas*, Psy—*Psychrobacter*, She—*Shewanella*, Aci—*Acinetobacter*, Fla—*Flavobacterium*, Agr—*Agrococcus*, Art—*Arthrobacter*, Bra—*Brachybacterium*, Cry—*Cryobacterium*, Lei—*Leifsonia*, Mic—*Micrococcus*, Pae—*Paeniglutamicibacter*, Rho—*Rhodococcus*, Sal—*Salinibacterium*, Fac—*Facklamia*, Spo—*Sporosarcina*, Car—*Carnobacterium*). (B) Loading plot of GC FA data. PC1—44% explained variance, PC2—23% explained variance. FA data were autoscaled before PCA. PUFAs (summed polyunsaturated fatty acids), n‐SFAs (summed non‐branched saturated fatty acids), br‐SFAs (summed branched saturated fatty acids), n‐MUFAs (summed non‐branched monounsaturated fatty acids), hydroxy‐FAs (summed hydroxy fatty acids), Cyclic‐FAs (summed cyclic fatty acids).

PCA of the GC data showed a clear distribution of the samples in the first principal component (PC1) mainly according to Gram groups and it was associated with the content of br‐SFAs/n‐SFAS/n‐MUFAs (Figure 2A). The majority of Gram‐positive bacteria clustered together mainly due to the presence of br‐SFAs and unknown FAs. Genus *Rhodococcus* from phylum Actinobacteria and genera *Facklamia* and *Carnobacterium* from phylum Firmicutes were grouped with Gram‐negative bacteria since they had n‐MUFAs as a major group of FAs (Figure 2A). The loading plot showed that the dissimilarities in the production of cyclic and hydroxy FAs in some *Pseudomonas* strains were responsible for the differences observed along the second principal component (PC2) axis (Figure 2B). Moreover, the PC1 axis shows differences caused by the temperature (Figure 2A), which were more apparent for Actinobacteria than for Firmicutes and Proteobacteria. For example, among Actinobacteria genera, *Cryobacterium* strains grown at different temperatures were clustered separately from each other, and *Arthrobacter* and *Leifsonia* strains cultivated at 5°C clustered separately from overlapping strains grown at 15°C and 25°C (Figure 2A). Temperature‐based clustering was also observed for some Proteobacteria genera, for example, *Shewanella* and *Pseudomonas*, but it was more species‐specific, and overlapping between different species can be seen. Also, it could be seen from the loading plot that PUFAs did not play a significant role in the clustering along PC1 (Figure 2B).

To deeply assess the main taxonomy‐aligned similarities and differences in overall fatty acid profile as well as temperature‐induced changes, fatty acid GC data were categorized into several groups according to (1) fatty acid chain length, including short‐chain fatty acids (SCFAs) containing less than 6 carbon atoms, medium‐chain fatty acids (MCLFAs) containing 7–12 carbon atoms, long‐chain fatty acids (LCFAs) containing 13–21 carbon atoms and very long‐chain fatty acids (VLCFAs) containing 22–24 carbon atoms, (2) fatty acid structural characteristics, including PUFAs (polyunsaturated fatty acids), n‐SFAs (non‐branched saturated fatty acids), br‐SFAs (branched saturated fatty acids), n‐MUFAs (non‐branched monounsaturated fatty acids), hydroxy‐FAs (hydroxy fatty acids) and cyclic‐FAs (cyclic fatty acids), (3) geometric isomerism (cis‐/trans‐FA) and (4) type of branching (iso‐/anteiso‐FA). Only FAs with content higher than 1% were included in the analysis.

The analysis of the fatty acids' chain length profile revealed that LCFAs containing 13–21 carbon atoms are the most common type of FAs (60%–98%) present in the studied Antarctic bacteria (Table 2). MCLFAs containing 7–12 carbon atoms were present in a relatively small amount (3%–11%) in Proteobacteria from genera *Pseudomonas*, *Acinetobacter*, *Shewanella* and in Bacteroidetes from genus *Flavobacterium*, where the highest amount was observed for *Pseudomonas* and *Flavobacterium* strains and it increased with the increase of the growth temperature. VLCFAs containing 22–24 carbon atoms were present in a small amount as well (up to 7%) in Actinobacteria from genera *Rhodococcus*, *Micrococcus*, *Brachybacterium*, all Firmicutes bacteria and Proteobacteria from genus *Acinetobacter*. The amount of VLCFAs increased at elevated cultivation temperatures (15°C or 25°C) for *Rhodococcus* and decreased for other genera (Table 2). SCFAs containing less than six carbon atoms were not detected in noteworthy amounts in the studied bacteria.

**TABLE 2:** The profile of different groups of FAs with varying chain lengths in Antarctic bacteria grown at different temperatures.

Gram	Genus	SCFAs			MCFAs			LCFAs			VLCFAs		
		5°C	15°C	25°C	5°C	15°C	25°C	5°C	15°C	25°C	5°C	15°C	25°C
Gram‐negative	Proteobacteria									
	Pol	0	0.03	‐	0.39	0.4	‐	98.93	96.2	‐	0.43	0.58	‐
	Pse	0.02 ± 0.01	0.02 ± 0.02	0.06 ± 0.08	8.87 ± 1.02	10.10 ± 1.44	11.31 ± 2.79	86.83 ± 2.14	85.79 ± 2.14	85.27 ± 3.77	1.82 ± 1.34	1.58 ± 1.23	0.92 ± 0.49
	Psy	0.03 ± 0.02	0.03 ± 0.02	0.07	0.40 ± 0.07	0.48 ± 0.19	0.39	97.87 ± 0.25	97.80 ± 0.73	97.59	0.72 ± 0.29	1.10 ± 0.97	1.14
	She	0.01 ± 0.01	0.01 ± 0.00	0.21 ± 0.09	3.28 ± 0.12	3.32 ± 0.09	2.85 ± 0.10	62.18 ± 0.95	54.72 ± 1.34	43.34 ± 1.34	1.69 ± 1.22	1.17 ± 0.82	0.76 ± 0.07
	Aci	0.05	0.11	0.44	5.76	5.28	7.9	89.31	89.52	88.29	4.29	3.35	1.25
	Bacteroidetes									
	Fla	0.02	0	‐	3.26	10.56	‐	73.62	72.22	‐	0.92	1.63	‐
Gram‐positive	Actinobacteria												
	Agr	0.08	‐	0.02	0.27	‐	0.29	63.19	‐	88.41	1.93	‐	1.29
	Art	0.03 ± 0.02	0.02 ± 0.01	0.02 ± 0.02	0.36 ± 0.07	0.34 ± 0.13	0.35 ± 0.11	86.89 ± 4.02	90.97 ± 3.56	90.12 ± 3.01	0.80 ± 0.36	0.73 ± 0.48	0.76 ± 0.41
	Bra	‐	0.32	0.02	‐	0.64	2.45	‐	78.25	76.26	‐	7.93	1.33
	Cry	0.05 ± 0.02	0.02 ± 0.02	0.04 ± 0.02	0.46 ± 0.12	0.43 ± 0.23	0.33 ± 0.07	78.63 ± 1.56	90.32 ± 2.58	96.35 ± 0.61	0.56 ± 0.15	0.51 ± 0.30	0.97 ± 0.35
	Lei	0.05 ± 0.02	0.04 ± 0.02	0.03 ± 0.02	1.35 ± 0.12	2.05 ± 0.23	0.88 ± 1.78	77.60 ± 1.56	84.65 ± 2.58	94.65 ± 2.86	1.17 ± 0.15	0.73 ± 0.30	0.77 ± 0.33
	Mic	0.04	0.05	0.03	0.71	0.56	0.52	84.98	80.47	69.78	4.84	1.63	3.08
	Pae	0.01	0.03	‐	0.26	0.43	‐	77.77	86.76	‐	0.36	0.35	‐
	Rho	0.03 ± 0.04	0.03 ± 0.02	0.03 ± 0.00	1.44 ± 0.41	1.17 ± 0.18	0.89 ± 0.14	94.20 ± 2.65	92.30 ± 5.11	90.86 ± 3.86	3.38 ± 2.70	5.08 ± 4.93	7.51 ± 3.56
	Sal	0.05	0.02 ± 0.02	0.08	3.13	3.15 ± 2.52	0.63	63.66	83.35 ± 6.04	85.39	0.44	0.93 ± 0.92	2.22
	Firmicutes												
	Fac	‐	0.08	‐	‐	0.72	‐	‐	87.89	‐	‐	7.16	‐
	Spo	0.12	0.03	0.05	0.69	0.48	0.39	89.42	88.37	64.61	5.31	3.02	2.77
	Car	0.03 ± 0.00	0.13 ± 0.17	0.38	0.62 ± 0.04	1.10 ± 0.76	0.5	92.13 ± 1.76	87.69 ± 11.21	97.12	3.07 ± 1.31	1.54 ± 0.56	0.84

*Note*: The standard deviation was calculated for genera that were represented by two or more strains. ‘‐’—no growth or not enough biomass for analysis .

Abbreviations: LCDA, long‐chain fatty acid; MCFA, medium‐chain fatty acid; SCFA, short‐chain fatty acid; VLCFA, very long‐chain fatty acid.

**FIGURE 3:**
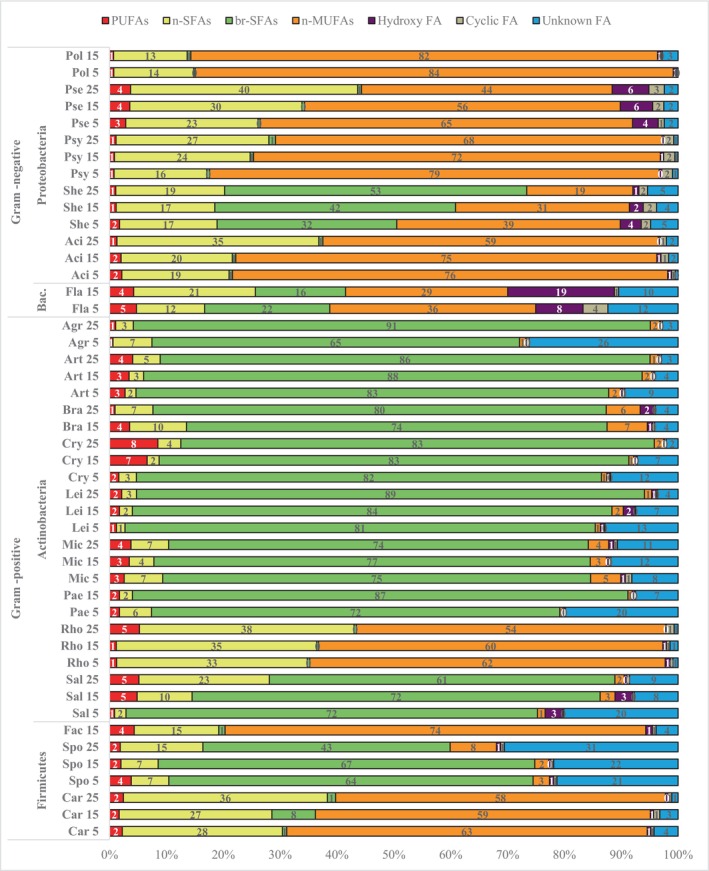
Fatty acid (FA) profile of bacteria grown at different temperatures (%, w/w), *—no growth/not enough biomass to perform analysis. Group of Fas: PUFAs (summed polyunsaturated fatty acids), n‐SFAs (summed non‐branched saturated fatty acids), br‐SFAs (summed branched saturated fatty acids), n‐MUFAs (summed non‐branched monounsaturated fatty acids), hydroxy‐FAs (summed hydroxy fatty acids), Cyclic‐FAs (summed cyclic fatty acids).

**FIGURE 4:**
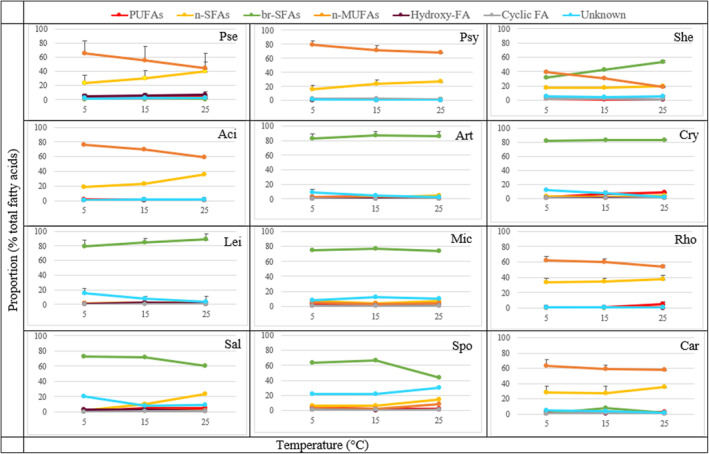
Fatty acid (FA) profile of Antarctic bacteria grown at varying growth temperatures. FAs with content lower than 1% were summed in “Minor FA”. *—FAs identified by GC–MS. GC–MS, gas chromatography–mass spectrometry.

The analysis of fatty acid profile based on the structural characteristics showed that all Gram‐positive bacteria, except *Rhodococcus* from phylum Actinobacteria as well as *Facklamia* and *Carnobacterium* from *the* phylum Firmicutes had branched fatty acids (br‐SFAs) as predominant ones (Figures 3 and 4 and Figures S3–S5 in the Supporting Information). Interestingly, all Gram‐positive bacteria were grouped into two groups according to their temperature‐induced changes of br‐SFAs: Actinobacteria from genera *Agrococcus*, *Arthrobacter*, *Brachybacterium*, *Cryobacterium*, *Leifsonia* and *Paeniglutamibacter* showed a continuous increase in br‐SFAs with elevating growth temperature, while *Salinibacterium* and *Sporosarcina* from phylum Firmicutes exhibited the opposite response (Figures 3 and 4 and Figures S3–S5 in the Supporting Information).

All Proteobacteria, except *Shewanella*, had straight‐chain monounsaturated fatty acids (n‐MUFAs) and non‐branched saturated fatty acids (n‐SFAs) as a major group of FAs at all studied temperatures (Figures 3 and 4 and Figures S3–S5 in the Supporting Information). Bacteria from genera *Shewanella* and *Flavobacterium* from phyla Proteobacteria and Bacteroidetes, respectively, had br‐SFAs present in their profile, which were not detected for other Gram‐negative bacteria. For all Gram‐negative bacteria, the quantity of n‐MUFAs was increasing with the temperature decrease, and the highest quantity was detected at 5°C. The quantity of n‐SFAs in Gram‐negative bacteria increased with temperature and reached maxima at 25°C (Figures 3 and 4 and Figures S3–S5 in the Supporting Information). *Shewanella* strains had n‐MUFAs as major FAs when grown at 5°C, and br‐SFAs when grown at 15°C and 25°C, respectively. No effect of temperature was detected for *Polaromonas* genus. Interestingly, similar fatty acid profiles and temperature responses were observed between Proteobacteria and Actinobacteria from the genus *Rhodococcus* and Firmicutes from genera *Facklamia* and *Carnobacterium* (Figures 3 and 4 and Figures S3–S5 in the Supporting Information).

A small amount of hydroxy‐FA was recorded in Antarctic bacteria and mostly for some Gram‐negative bacteria from Proteobacteria and Bacteroidetes phyla. For example, Proteobacteria from the genus *Pseudomonas* and Bacteroidetes from the genus *Flavobacterium* were characterized by higher OH‐FA production at higher growth temperatures (15°C and 25°C), up to 17% and 19%, respectively (Figures 3 and 4 and Figures S3–S5 in the Supporting Information). Some Proteobacteria strains produced PUFAs, for example, *Pseudomonas* sp. BIM B‐1674 produced up to 18% of PUFAs of the total fatty acid content, and *P. lundensis* BIM B‐1554 produced up to 10% of PUFAs when grown at 15°C and 25°C, respectively (Figures 3 and 4 and Figures S3–S5 in the Supporting Information). Interestingly, for the genus *Cryobacterium*, it was detected an increase in the amount of PUFAs was with the increase of growth temperature (Figures 3 and 4 and Figures S3–S5 in the Supporting Information). Small amounts of cyclic‐FAs produced by Proteobacteria from the genus *Pseudomonas* increased with the increase in temperature. For example, *Pseudomonas* sp. BIM B‐1674 produced up to 8% of cyclic‐FAs (Figures 3 and 4 and Figures S3–S5 in the Supporting Information).

Distinct patterns in fatty acid profiles and temperature impact were observed for psychrophiles and psychrotrophs. Thus, fatty acid profiles of *Polaromonas*, *Carnobacterium* and *Rhodococcus* psychrophilic strains remained unchanged at 5°C and 15°C of cultivation. In contrast, the fatty acid profile of the psychrophilic *Flavobacterium* and *Psychrobacter* strains was influenced by temperature, leading to an increase in n‐SFAs and a decrease in n‐MUFAs at 15°C compared to 5°C. For psychrotrophic strains from *Paeniglutamibacter* genera, the proportion between br‐SFAs and unknown FAs decreased at 5°C compared to 15°C (Figures S3 and S4 in the Supporting Information).

**FIGURE 5:**
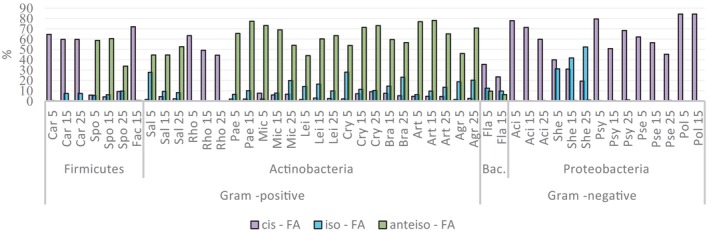
The amount of cis‐, iso‐ and anteiso‐ FAs of Antarctic bacteria grown at varying growth temperatures. FA, fatty acid.

All Gram‐negative bacteria were characterized by the production of cis‐fatty acids (cis‐FAs). *Shewanella* and *Flavobacterium* are characterized by the lowest amount of cis‐FAs among all studied bacteria. Among Gram‐positive bacteria, the production of cis‐FAs was detected in both phyla, in phyla Actinobacteria for genus *Rhodococcus* and in phyla Firmicutes for *Carnobacterium* and *Facklamia* (Figure 5 and Figures S6–S8 in the Supporting Information). The presence of trans‐fatty acids (trans‐FAs) was detected in small quantities (data not shown). A noticeable increase in the synthesis of cis‐FAs along with the growth temperature decrease was noted for some Proteobacteria, especially for species of *Pseudomonas*, *Shewanella* and *Acinetobacter*, as well as for *Flavobacterium* from phylum Bacteroidetes (Figure 5 and Figures S6–S8 in the Supporting Information). In contrast, the profile of *Polaromonas* remained unchanged regarding cis‐isomerization (Figure 5 and Figures S6–S8 in the Supporting Information). Among Gram‐positive bacteria, an increase in the production of cis‐FAs was detected in *Rhodococcus* and *Carnobacterium* from phyla Actinobacteria and Firmicutes, respectively.

Anteiso‐FAs were more characteristic for Actinobacteria and for *Sporosarcina* from phylum Firmicutes. Iso‐FAs were detected in small amounts in all Actinobacteria, Firmicutes, and Bacteroidetes phyla and in Proteobacteria only genus *Shewanell*a was characterized by the production of this type of FA. Mainly, genus‐specific temperature‐induced changes were observed, for example, for the genera *Sporosarcina*, *Micrococcus* and *Arthrobacter*, it decreased with the temperature decrease. For the genera *Paeniglutamicibacter*, *Leifsonia*, *Cryobacterium* and *Agrococcus*, the opposite effect was observed. A clear increase in the amount of iso‐FAs was detected for the genera *Shewanella*, *Agrococcus*, *Arthrobacter*, *Brachybacterium*, *Micrococcus* and *Paeniglutamicibacter* with an increase in growth temperature, while *Salinibacterium* showed an opposite response (Figure 5 and Figures S6–S8 in the Supporting Information).

When comparing the ratios of cis‐/trans‐ and iso‐/anteiso‐FAs in psychrophilic and psychrotrophic bacteria, it can be seen that psychrophilic Gram‐negative Proteobacteria genus *Polaromonas* possess little change in the cis‐/trans‐FAs ratio, with cis‐FAs being the predominant type. However, for bacteria from the genus *Psychrobacter*, the production of trans‐FAs was detected at 15°C and not at 5°C (Figures S6 and S7 in the Supporting Information). Gram‐positive Actinobacteria showed genus‐specific changes similar to psychrotrophic bacteria. Thus *Leifsonia* strains had an increase in iso‐FAs and a decrease in anteiso‐FAs with temperature downshift. Interestingly, trans‐FAs were not detected at 15°C in psychrophilic *Rhodococcus erythropolis* BIM B‐1661 similarly as for psychrotrophic *Rhodoccocus* strains (Figures S6 and S7 in the Supporting Information).

**FIGURE 6:**
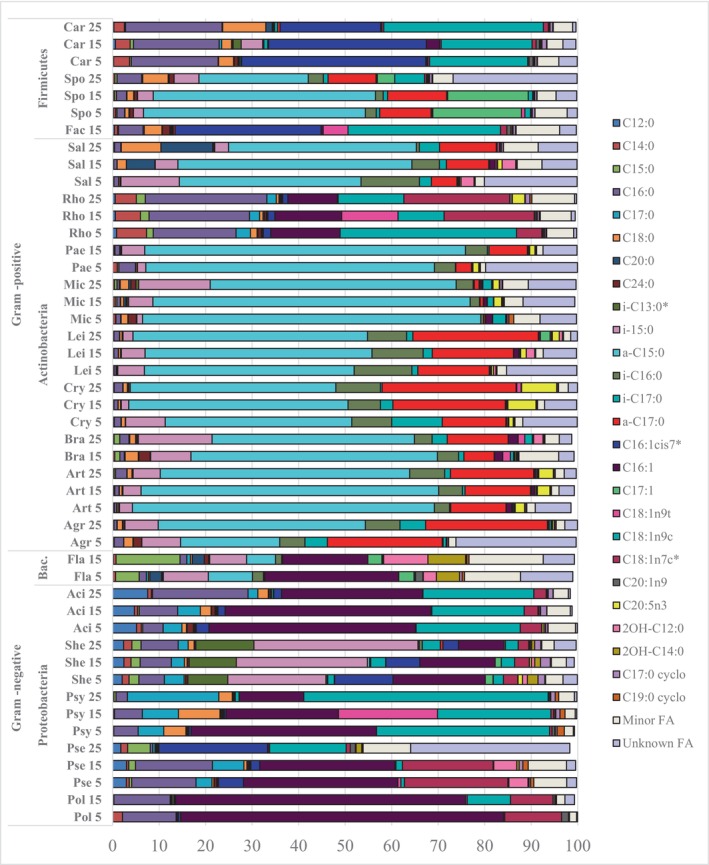
The effect of temperature on fatty acid (FA) classes in bacteria cultivated at 5°C, 15°C and 25°C (mean ± SD). The standard deviation was calculated for genera that were represented by two or more strains. Group of FAs: PUFAs (summed polyunsaturated fatty acids), n‐SFAs (summed non‐branched saturated fatty acids), br‐SFAs (summed branched saturated fatty acids), n‐MUFAs (summed non‐branched monounsaturated fatty acids), hydroxy‐FAs (summed hydroxy fatty acids), Cyclic‐FAs (summed cyclic fatty acids); Genera: Pse—*Pseudomonas*, Psy—*Psychrobacter*, She—*Shewanella*, Aci—*Acinetobacter*, Art—*Arthrobacter*, Cry—*Cryobacterium*, Lei—*Leifsonia*, Mic—*Micrococcus*, Rho—*Rhodococcus*, Sal—*Salinibacterium*, Spo—*Sporosarcina*, Car—*Carnobacterium*.

Using the GC fatty acid profile, we tried to identify the most predominant fatty acids for different taxonomic groups, and we observed genera‐specific differences. Thus, for Proteobacteria from genera *Polaromonas* and *Pseudomonas*, a similar fatty acid profile with C16:1, C18:1n7c and C16:0 as predominant FAs was observed. Other genera from this phylum, such as *Psychrobacter* and *Acinetobacter*, share similar profiles, with C16:1 and C18:1n9c as predominant fatty acids, and *Psychrobacter* also having C17:0 as a third dominant fatty acid, while *Acinetobacter* has C16:0 as a third dominant fatty acid. *Shewanella* stands out with the most distinct profile compared to other genera from phylum Proteobacteria and it was characterized by i‐C15:0, C16:1 and i‐C13:0 as predominant fatty acids. Bacteroidetes phylum was represented by one genus *Flavobacterium* which showed a distinct fatty acid profile sharing some similarities with Gram‐positive and Gram‐negative, and additionally, we observed other FAs in significant amounts, such as C15:0 and 2OH‐C14:0 (Figure 6). Among Gram‐positive bacteria, the majority of Actinobacteria, except for *Rhodococcus*, *Paeniglutamicibacter* and *Micrococcus*, exhibit similar fatty acid profiles with predominant FAs a‐C15:0 and a‐C17:0 (Figure 6). However, *Paeniglutamicibacter* and *Micrococcus* have a lower quantity of a‐C17:0. On the other hand, *Rhodococcus* displays a completely different profile, resembling the profile of Proteobacteria from genera *Pseudomonas*, *Polaromonas*, and *Acinetobacter*. Among Firmicutes, *Carnobacterium* and *Facklamia* have similar profiles, with C16:1cis7 and C18:1n9c being predominant FAs that also differs from *Sporosarcina*, which has predominant FAs a‐C15:0 and C17:1 (Figure 6).

**FIGURE 7:**
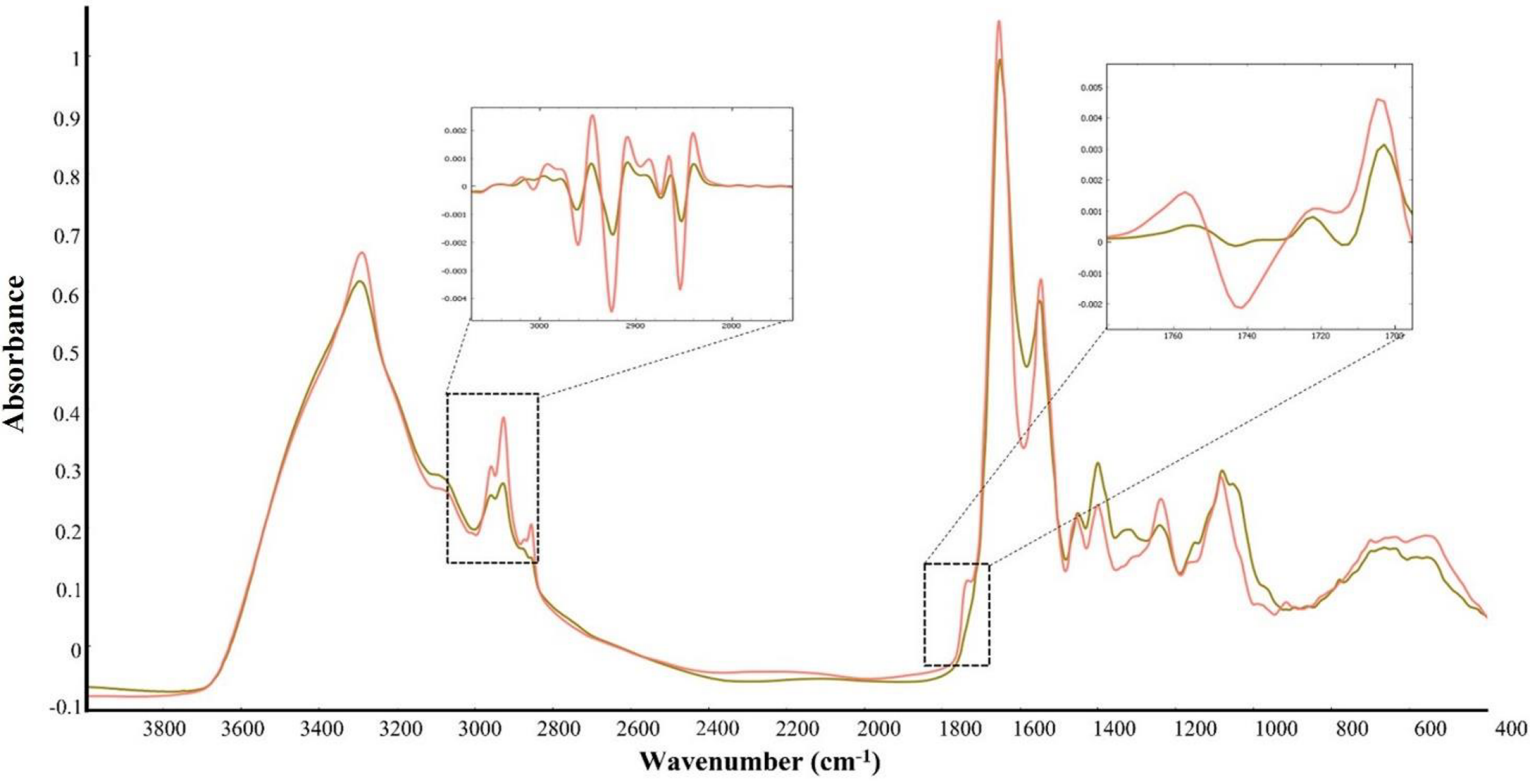
EMSC corrected FTIR spectra of two bacteria grown at 15°C with different total lipid content (%): olive—*Brachybacterium paraconglomeratum* BIM B—1571 (4%), orange—*Pseudomonas peli* BIM B—1546 (18%). EMSC, extended multiplicative signal correction; FTIR, Fourier transforms infrared.

**FIGURE 8:**
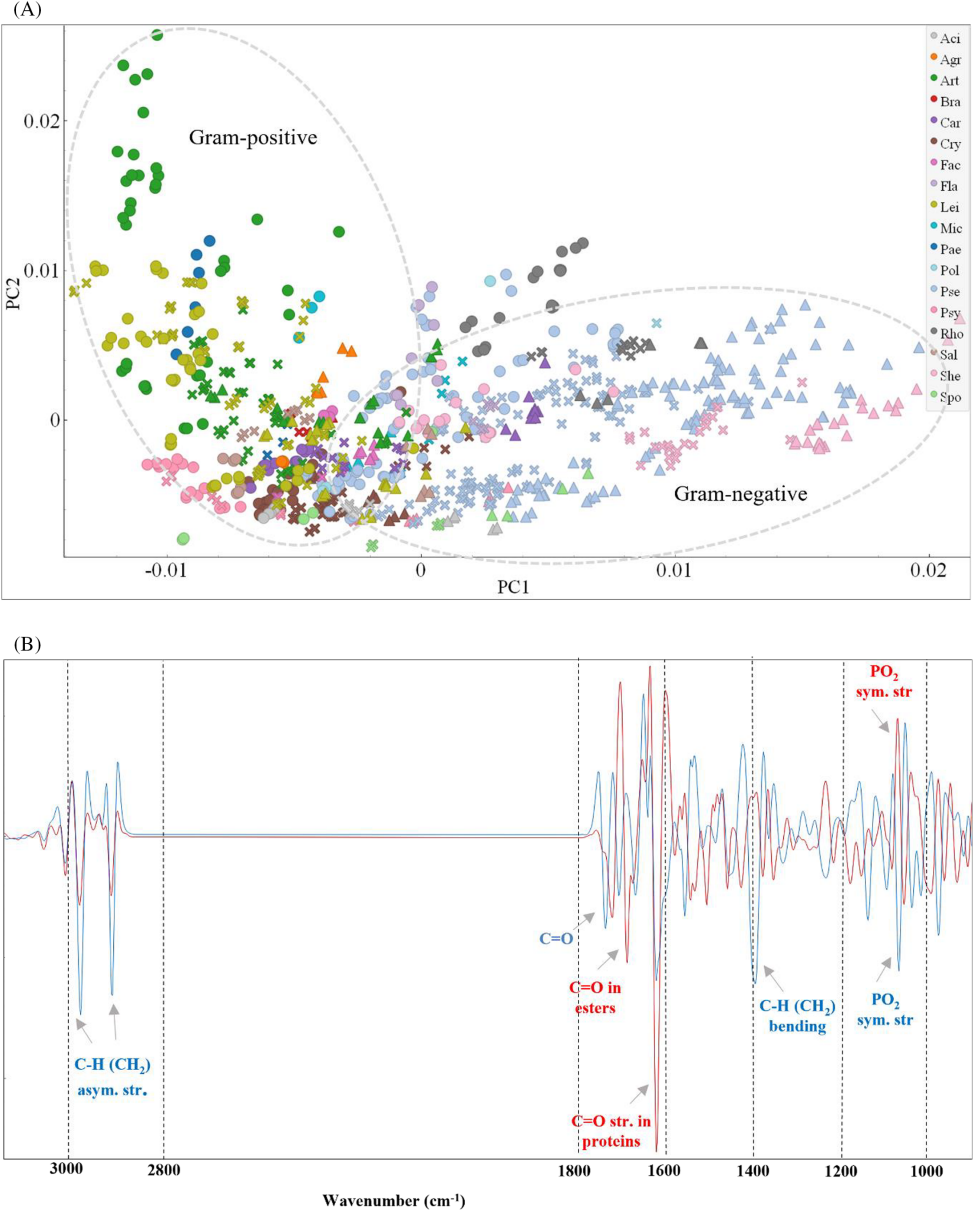
PCA of the preprocessed FTIR spectra of Antarctic bacteria grown at different temperatures (‘●’—5°C, ‘✖’—15°C and ‘▲’—25°C). (A) Score plot of PC1 and PC2 components, colours represent genera, shapes represent cultivation temperatures, different colours represent genera (Pol—*Polaromonas*, Pse—*Pseudomonas*, Psy—*Psychrobacter*, She—*Shewanella*, Aci—*Acinetobacter*, Fla—*Flavobacterium*, Agr—*Agrococcus*, Art—*Arthrobacter*, Bra—*Brachybacterium*, Cry—*Cryobacterium*, Lei—*Leifsonia*, Mic—*Micrococcus*, Pae—*Paeniglutamicibacter*, Rho—*Rhodococcus*, Sal—*Salinibacterium*, Fac—*Facklamia*, Spo—*Sporosarcina*, Car—*Carnobacterium*). (B) Loading plot of FTIR data with the main contributing peaks, PC1 (red) and PC2 (blue). PC1 provided 35% of explained variance and PC2 provided 17% of explained variance. FTIR, Fourier transforms infrared.

**FIGURE 9:**
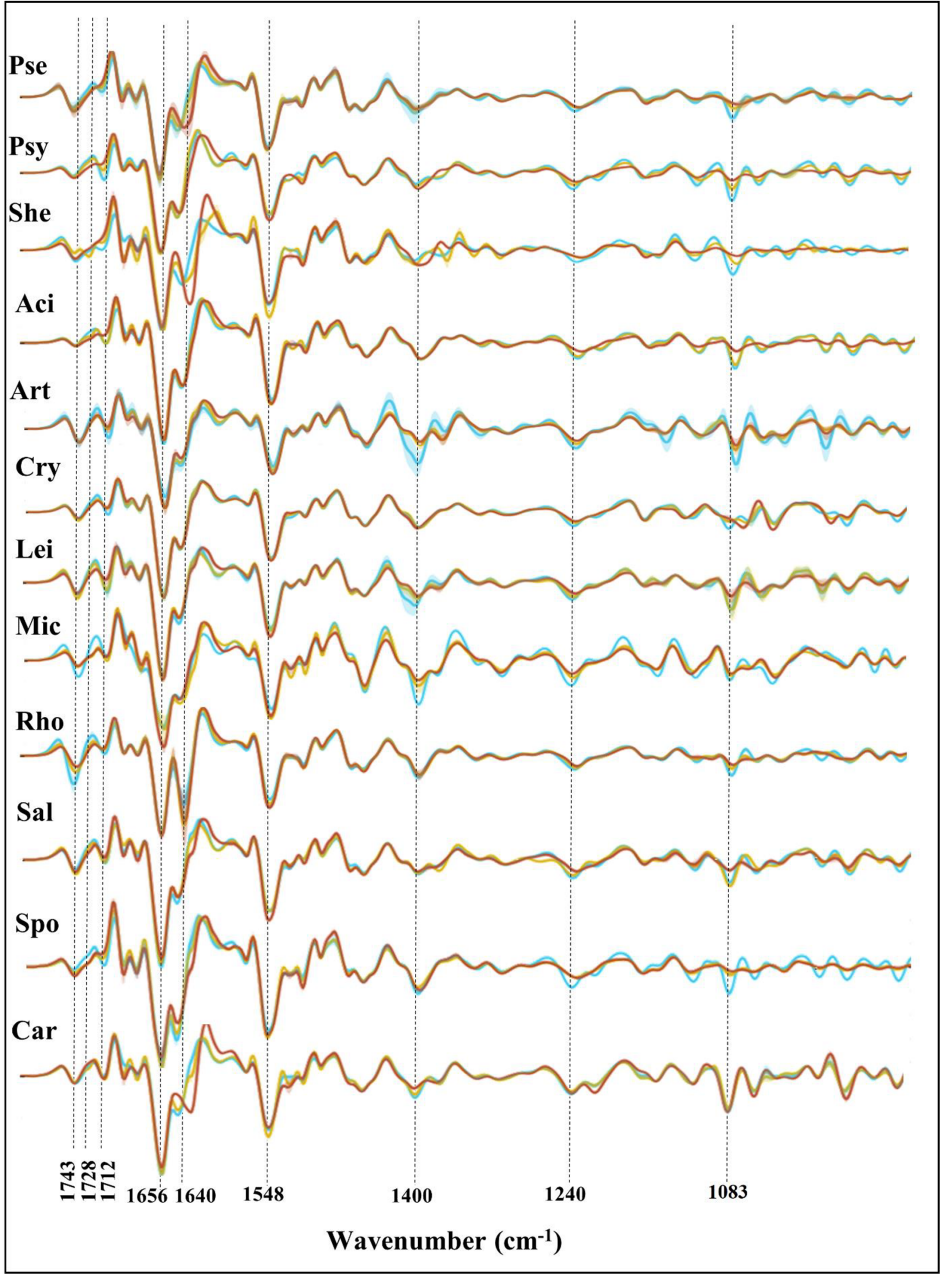
Second derivative FTIR spectra of bacterial biomass of different genera grown at different temperatures (blue—5°C, yellow—15°C and orange—25°C). Genera: Pse—*Pseudomonas*, Psy—*Psychrobacter*, She—*Shewanella*, Aci—*Acinetobacter*, Art—*Arthrobacter*, Cry—*Cryobacterium*, Lei—*Leifsonia*, Mic—*Micrococcus*, Rho—*Rhodococcus*, Sal—*Salinibacterium*, Spo—*Sporosarcina*, Car—*Carnobacterium*. FTIR, Fourier transforms infrared.

**FIGURE 10:**
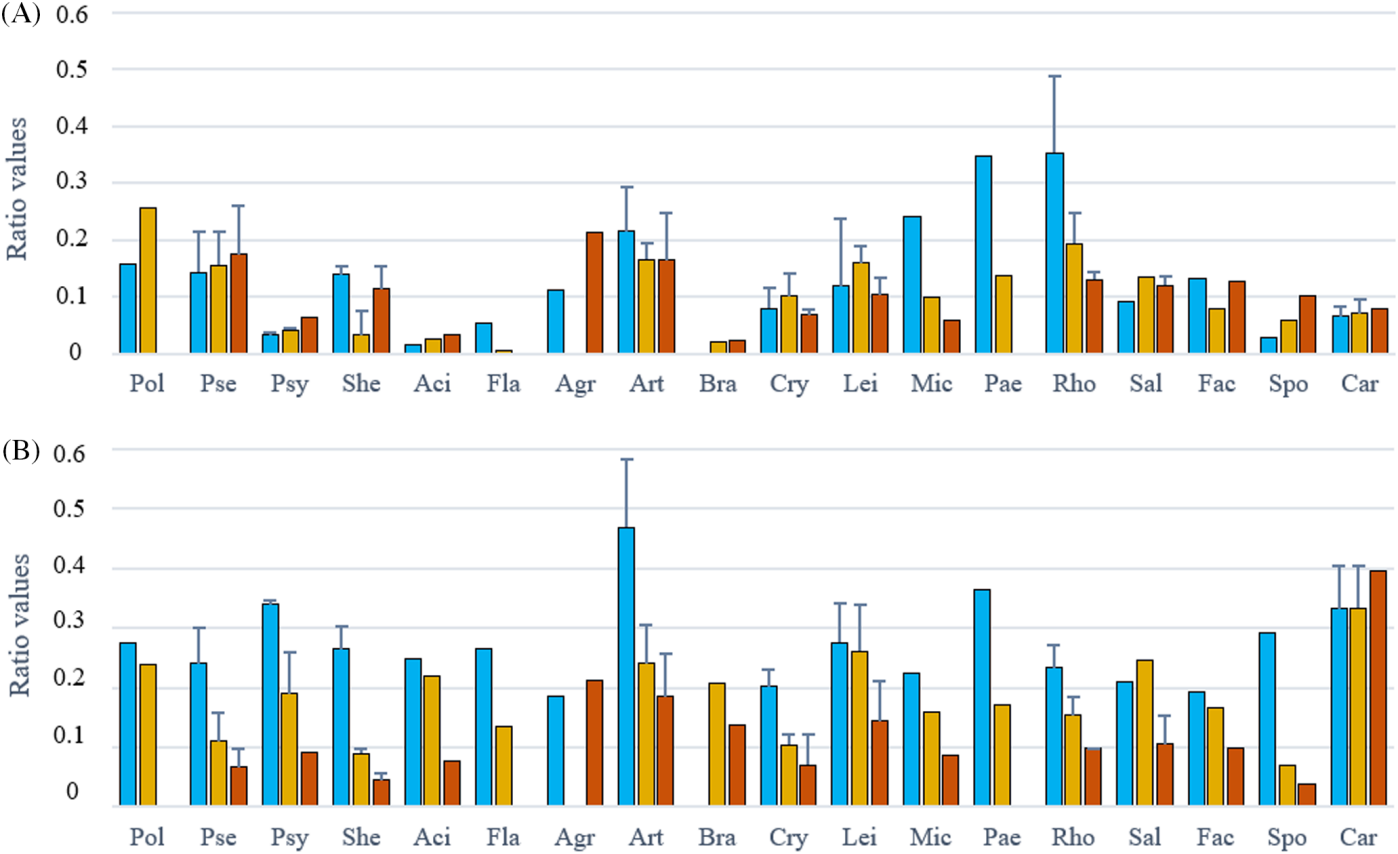
The relative content of the cellular components in bacterial biomass grown at different temperatures (blue—5°C, yellow—15°C and orange—25°C) estimated by ratio‐based analysis using FTIR spectra, where: A—lipid/protein ratio (1734/1656 cm^−1^) and B—phosphorus‐based compounds/protein ratio (1083/1656 cm^−1^). The standard deviation was calculated for genera that were represented by two or more strains. Genera: Pol—*Polaromonas*, Pse—*Pseudomonas*, Psy—*Psychrobacter*, She—*Shewanella*, Aci—*Acinetobacter*, Fla—*Flavobacterium*, Agr—*Agrococcus*, Art—*Arthrobacter*, Bra—*Brachybacterium*, Cry—*Cryobacterium*, Lei—*Leifsonia*, Mic—*Micrococcus*, Pae—*Paeniglutamicibacter*, Rho—*Rhodococcus*, Sal—*Salinibacterium*, Fac—*Facklamia*, Spo—*Sporosarcina*, Car—*Carnobacterium*. FTIR, Fourier transforms infrared.

For Gram‐positive bacteria, some FAs were not identified with the use of external standards and the GC–MS library. The amount of these FAs increased with the decrease in growth temperature, so it can be assumed that these unknown FAs belong to a group of unsaturated unbranched FAs or unsaturated branched FAs (Figures 3 and 4 and Figures S3–S5 in the Supporting Information).

Strain *Leifsonia antarctica* BIM B‐1671 showed a fatty acid profile distinctly different from all other *Leifsonia* strains (Figure S3–S5 in the Supporting Information). This and other previously reported considerable similarities of the total cellular biochemical profile of this strain with *Pseudomonas* (Smirnova et al., 2021; Smirnova et al., 2022) can be an indication of misidentification by 16S rRNA gene sequencing.

### Impact of temperature on the total cell chemistry

Intact bacterial biomass obtained from the cultivation at different temperatures was analysed by the HTS‐FTIR spectroscopy for evaluating changes in main cellular biomolecules, such as lipids, proteins, and polysaccharides. Figure 7 shows the representative FTIR spectra of two Antarctic bacteria with low and high lipid content. In Figure 7, the primary spectral regions associated with lipids are 3100–2800 cm^−1^ (C‐H), which indicates the presence of fatty acid chains in lipids, and 1800–1700 cm^−1^ (C=O), which indicates the presence of triacyl glycerides, free fatty acids, or polyesters. The observed changes in these peaks exhibit a strong correlation with the changes in the total lipid content that was measured using GC–FID.

Preprocessed FTIR spectra were analysed by PCA, and score and loading plots are displayed in Figure 8. Along the PC1 axis, a clear separation resembling Gram‐groups and less clear for phylum‐ and genera‐based classification, and temperature effect could be seen (Figure 8A). Specifically, Gram‐negative Proteobacteria predominantly had positive PC1 scores, while Gram‐positive Actinobacteria predominantly had negative PC1 scores, and bacteria from phyla Bacteroidetes and Firmicutes overlap with each other and other phyla (Figure S10 in the Supporting Information). Furthermore, a clear separation along the PC1 axis was observed between *Shewanella*, *Pseudomonas* and *Acinetobacter* cultivated at different temperatures (Figure 8A). Both PC1 and PC2 appeared to be responsible for the dissimilarities between Gram‐positive bacteria cultivated at different temperatures. Thus, a clear separation between bacteria grown at 5°C and 25°C was observed for *Arthrobacter*, *Psychrobacter*, *Carnobacterium*, *Cryobacterium* and *Leifsonia* genera. The scores for bacteria cultivated at 15°C usually overlapped with scores for those cultivated at 5°C or 25°C (Figure 8A). The loading plots in Figure 8B illustrate the weight of each original variable (wavenumbers) on the PCs and the contribution of each spectral feature. The separation along the PC1 axis was due to changes in the C=O stretching peak (amide I) in proteins at 1627 cm^−1^, P‐O‐C symmetric stretching peak probably related to phospholipids at 1083 cm^−1^, and C=O stretching in esters and aldehydes at 1709 and 1725 cm^−1^. The separation along the PC2 axis can be explained by the changes in the C‐H (CH_2_) stretching in saturated lipids at 2924 and 2853 cm^−1^, C=O stretching of esters and aldehydes at 1742 cm^−1^, CH_2_ bending in lipids with little contributions from protein (membrane lipids) at 1400 cm^−1^, and P‐O‐C symmetric stretching peak probably related to phospholipids at 1083 cm^−1^ (Figure 8B).

The effect of temperature on the C=O stretching region (1800–1700 cm^−1^) was evaluated and the increase in absorbance for the ester peak at 1743 cm^−1^ along with the temperature decrease was observed for Gram‐positive Actinobacteria from genera *Micrococcus* and *Rhodococcus* (Figure 9). There was no temperature effect on the ester peak detected for Firmicutes and all Gram‐negative bacteria. An increase in the peak at 1712 cm^−1^ associated with C=O stretching in free FAs along with the decrease of cultivation temperature was detected for all studied bacteria except bacteria Firmicutes (Figure 9 and Figure S12 in the Supporting Information). Some genus‐specific changes were observed for Gram‐negative bacteria, where *Shewanella* showed significant changes associated with the absorbance decrease for the ester peak at 1745 cm^−1^ and the appearance of an additional peak at 1728 cm^−1^ at 15°C (Figure 9).

In the protein region (1700–1500 cm^−1^), the biggest effect of temperature was detected for the amide I peak at 1640 cm^−1^ related to β‐sheet structures of proteins, where an increase in absorbance was observed for the majority of the studied Antarctic bacteria grown at higher temperatures. Further, genus‐specific effect in the form of a shift to lower wavenumbers was recorded for Proteobacteria genera *Shewanella* and *Pseudomonas* and Firmicutes genus *Carnobacterium* at higher growth temperature (Figure 9). Bacteria from the genus *Rhodococcus* showed an equal amount of α‐helical (peak at 1656 cm^−1^) and β‐pleated sheet (peak at 1640 cm^−1^) structures, whereas α‐helical structures seem to dominate in all other bacteria (Figure 9). There was no effect of temperature detected for the amide II peak associated with the vibrations of N‐H plane amide groups at 1548 cm^−1^.

The most significant temperature‐triggered alterations were recorded in the mixed spectral region 1500–900 cm^−1^, where signals related to carbohydrates, nucleic acids, and phosphates are present. The effect of temperature on this region was considerable for all taxonomic levels (Figure 9 and Figures S11 and S12 in the Supporting Information). Thus, an increase in intensity for several peaks in the mixed region (1400, 1240 and 1083 cm^−1^) along with temperature decrease was recorded for the majority of Proteobacteria, Bacteroidetes and Actinobacteria, while changes for Firmicutes were less intense. The most significant changes were detected for the genera *Pseudomonas*, *Psychrobacter*, *Shewanella*, *Acinetobacter*, *Leifsonia*, *Rhodococcus* and *Salinibacterium*. Nearly all bacteria had changes in symmetric stretching peak at 1083 cm^−1^ which was significantly higher with temperature decrease (Figure 9).

An increase in absorbance for the peak at 1400 cm^−1^, related to ‐CH_2_ bending vibrations in lipids at lower cultivation temperatures was observed for Bacteroidetes and several genera of Actinobacteria such us *Arthrobacter*, *Leifsonia*, *Micrococcus* and *Carnobacterium* (Figure 9).

### Compositional analysis based on the estimation of ratios using FTIR spectra

To evaluate the effect of growth temperature and estimate the relative content of the main cellular components in bacterial biomass, several ratio‐based parameters were effectively estimated. The protein peak at 1654 cm^−1^ (amide I) could be considered a relatively stable component as can be seen in Figure 9 and it was used to estimate the relative content of lipids and phosphorus‐containing components in the same way as it was previously done for microalgae (Dean et al., 2010) and fungi (Dzurendova et al., 2021). The following ratio parameters were calculated: (1) lipid to protein ratio (L/P), allowing estimate relative total lipid content, was estimated by using ester bond C=O stretching peak at 1743 cm^−1^ and protein amide I peak at 1654 cm^−1^; (2) ratio of phosphorus‐containing components over proteins (P/P), determining the total content of phospholipids and to less extent nucleotides, was estimated using P‐O‐C symmetric stretching peak at 1083 cm^−1^, probably related to phospholipids and amid I peak at 1654 cm^−1^.

Overall, the highest L/P ratio was observed for Gram‐positive bacteria from Actinobacteria from genera *Rhodococcus* and *Paeniglutamicibacter*, and among Gram‐negative Proteobacteria from genera *Polaromonas* and *Pseudomonas*, while the lowest L/P ratio was detected for *Acinetobacter*, *Flavobacterium* and *Brachybacterium* from phyla Proteobacteria, Bacteroidetes and Actinobacteria, respectively (Figure 10). The L/P ratio for Actinobacteria genera *Rhodococcus*, *Paeniglutamicibacter*, *Micrococcus* and *Arthrobacter* and Bacteroidetes genus *Flavobacterium* was significantly increasing along with temperature decrease. An opposite effect was observed for all Proteobacteria except genus *Shewanella*, Firmicutes genera *Sporosarcina* and *Carnobacterium*, and Actinobacteria genera *Agrococcus* and *Salinibacterium*, where the L/P ratio was rising with temperature increase (Figure 10). Interestingly, the L/P ratio for *Shewanella* was lower at 15°C than at 5°C and 25°C (Figure 10). A significant effect of temperature on phospholipids and other phosphorus‐containing compounds was detected. Thus, for all Gram‐negative bacteria and the majority of Gram‐positive bacteria except *Agrococcus* and *Carnobacterium*, a significant increase in P/P ratio with temperature downshift was observed.

Pearson correlation coefficient (*r*) was calculated to examine the relationship between the L/P ratio and total lipid content (Figure S13 in the Supporting Information). The correlation coefficient for the entire dataset was found to be 0.36. When comparing Gram‐negative and Gram‐positive bacteria, a higher correlation coefficient was observed for Gram‐negative bacteria (*r* = 0.63) compared to Gram‐positive bacteria (*r* = 0.26) (Figure S13A in the Supporting Information). When comparing correlation for different phyla, Bacteroidetes had the highest correlation (1), then Proteobacteria (0.59), and then Firmicutes (0.56) and the lowest was for Actinobacteria (0.15). When comparing different genera within the Gram‐negative group, the highest correlation coefficients were found for *Psychrobacter* (0.59) and *Pseudomonas* (0.57). Among the Gram‐positive, the highest correlation coefficients were found for *Rhodococcus* (0.89), *Carnobacterium* (0.88), *Salinibacterium* (0.80) and *Leifsonia* (0.65), whereas the lowest coefficient was observed for *Cryobacterium* (0.13) (Figure S13C in the Supporting Information). Some genera showed weak positive or negative linear relationships: *Arthrobacter* (−0.05), *Cryobacterium* (0.13) and *Shewanella* (0.10). Further analysis revealed that the correlation coefficient decreased along with temperature decrease, where at 25°C, the coefficient was 0.71, at 15°C it was 0.44, and at 5°C it dropped to 0.09 (Figure S13B in the Supporting Information). The correlation within each genus varies for cultivation at different temperatures. However, for the majority of genera, there is an increase in correlation at higher temperatures (Figure S13D–G in Supporting Information).

## DISCUSSION

Microorganisms respond to changing environmental conditions by activating their adaptation mechanisms. Polar regions are extreme environments characterized by the presence of several stress factors, such as nutrient limitation, salinity, water availability, fluctuations in temperature and UV radiation (Rothschild & Mancinelli, 2001; Thomas & Dieckmann, 2002). Due to that, bacteria inhabiting polar regions may have unique adaptation mechanisms allowing them to survive and develop in these conditions (Barria et al., 2013; De Maayer et al., 2014; Mocali et al., 2017; Singh, 2022; Tribelli & López, 2018). Cold‐adapted bacteria have been extensively studied for decades, while most of the reported studies focus on very targeted biomolecules. The explorative characterization covering several biomolecules allowing us to obtain more comprehensive knowledge has not been previously performed for cold‐adapted bacteria. This study reports, for the first time, comprehensive taxonomy‐aligned characterization of the total cellular biomolecules profile (lipids, proteins and polysaccharides) for 74 Antarctic bacteria isolated from green snow and meltwater ponds. In addition, we show what changes occur for different cellular biomolecules when these bacteria grow at different temperatures and how these alterations vary for different taxonomic groups. Important to highlight that the set of bacteria used in the study is not balanced according to different taxonomic units. For example, nine genera were represented by only one species, phylum Bacteroidetes was represented by one and Firmicutes by five species. Therefore, comparison of the achieved results on phylum and genus level is limited by this set of bacteria and could not be used to draw any general conclusions. In the case of Gram‐groups, we had quite a balanced distribution where 44 strains were Gram‐positive, and 30 strains were Gram‐negative; therefore, comparison according to Gram can be used to draw a general hypothesis.

Research on bacteria from the Antarctic snow and meltwater pounds is important for the prediction of future climate‐associated changes in this region. Extensive formation of meltwater pounds in Antarctica results in a higher absorption of solar energy due to the dark colour of the meltwater ponds which may lead to a quicker heat transfer to soil (Perovich et al., 2002; Stokes et al., 2019). Soil in Antarctica and other polar and alpine regions exhibit notable heterogeneity of bacterial communities which play a significant role in these environments (Wiebe et al., 1992). An increase in the appearance of the meltwater ponds and their long‐term existence due to climate change and longer summers may lead to a change in soil microbiota.

Previously, it has been shown that the majority of Antarctic bacteria are psychrotrophic (Ray et al., 1998) which was also observed in this study. Psychrotrophic bacteria are well‐adapted to cold environments but can also survive and function at moderate temperatures (Ilicic et al., 2023). A previous study proposed that key factors influencing microbial distribution in Antarctic ecosystems are temperature and nutrient availability, where increasing temperature potentially stimulates bacterial growth (Wiebe et al., 1992). However, opposite results have also been reported (Hodson et al., 1981). In our study, we demonstrate that the majority of isolated Antarctic bacteria can thrive across a wide range of temperatures, from 4°C to 30°C and even 37°C, showing their extraordinarily high metabolic plasticity.

To perform biomolecular characterization of the studied Antarctic bacteria grown at different temperatures we have selected a BHI broth medium. Despite the differences in growth characteristics between BHI agar and BHI broth, BHI broth nutrient‐rich medium was selected due to its ability to provide a well‐mixed and uniform environment for bacterial growth and to mitigate the impact of nutrient limitations (Bonnet et al., 2020). This medium was effective in supporting the growth of all studied bacteria as well as evaluating the overall temperature's effect on total lipid content, fatty acid profile, and total cellular biochemical profile as was previously reported (Smirnova et al., 2021; Smirnova et al., 2022), while to identify potentially oleaginous bacteria, high C/N media are necessary to use. This medium was also chosen due to the lack of lipids since they could potentially affect the fatty acid profile of bacteria, as many bacteria can incorporate lipids into their cell membranes (Yao & Rock, 2017). Studied Antarctic bacteria are fast‐growing as was previously reported (Akulava et al., 2022; Smirnova et al., 2021); therefore, we cultivated them for 7 days until they reached the stationary phase. According to the literature, the biggest differences in cell chemistry are happening between lag, log, and stationary phases, and the stationary phase is considered the most chemically stable (Kochan et al., 2020). It was also shown in previous works on filamentous fungi that after 3 days of fermentation the fatty acid composition stabilizes (Kosa, Kohler, et al., 2017).

It has been previously reported that alterations in the total lipid content of bacterial cells, as well as their fatty acid composition, are one of the main adaptation mechanisms to continuously changing temperature conditions. Because fatty acid composition is used as an important biomarker for identifying, classifying and differentiating closely related bacterial species (Sasser, 1990), the alterations in lipids are often taxonomy‐specific and differ from genus to genus and species to species. Determining temperature‐associated changes of lipids in bacteria is particularly important for gaining insights into their physiology, diversity (De Carvalho & Caramujo, 2014), resistance mechanisms (Dunnick & O'Leary, 1970), and taxonomic relationships.

In this study, we observed that total lipid content and its alterations triggered by temperature changes are mainly species‐specific, and they can vary considerably. For example, total lipid content in *Pseudomonas* species varied from 6%_w/w_ to 19%_w/w_. Further, a clear difference in the total lipid content between genera of the same phylum was recorded, where genera *Pseudomonas* and *Shewanella* from the phylum Proteobacteria were characterized by the highest lipid production. For example, *Pseudomonas peli* strains had lipids from 12%_w/w_ to 19%_w/w_, depending on the strain and growth temperature. Such a high lipid content in *Pseudomonas peli* was not reported previously, according to the authors' knowledge, and it can be also explained by the possible production of polyesters (Röttig & Steinbüchel, 2016). For *Pseudomonas leptonychotis*, total lipid content ranged from 12%_w/w_ to 14%_w/w_, and for *Shewanella baltica* from 10%_w/w_ to 12%_w/w_, which was consistent with other *Shewanella* strains described in the literature (Zhang & Burgess, 2017). Overall, the obtained results are in accordance with the previously reported and can be explained by the fact that Gram‐positive bacteria have naturally higher peptidoglycan content, whereas Gram‐negative bacteria have higher lipid content (Feijó Delgado et al., 2013; Tripathi & Sapra, 2020). Gram‐negative bacteria have an outer membrane, in addition to their inner membrane, which is composed of lipopolysaccharides (LPS) and phospholipids that can contribute to the higher lipid content. Interestingly, in our study, we observed that certain Gram‐negative bacteria possess low lipid amounts comparable to or even lower than those found in Gram‐positive bacteria, as it was for *Polaromonas*, *Psychrobacter* and *Acinetobacter*. An increase in total lipid content with temperature decrease was detected for the majority of bacteria tested except for some *Arthrobacter* and *Pseudomonas* species. This adaptation mechanism was previously shown by other researchers (Hunter et al., 1981).

Fatty acid profiling by GC–FID and GC–MS indicated Gram‐ and taxon‐related differences in the fatty acid composition for the studied Antarctic bacteria. Overall, the obtained results correlated well with the previously reported (Bajerski et al., 2017; Hassan et al., 2020; Mező et al., 2022; Zhang & Rock, 2008). Thus, most of the Gram‐positive bacteria were characterized by a high content of br‐SFAs, except *Rhodococcus*, *Facklamia* and *Carnobacterium*, while Gram‐negative bacteria were characterized by a high content of n‐MUFAs, except *Shewanella*; these observations are in accordance with previously reported results (Garba et al., 2016).

Chain length in phospholipid tails impacts membrane fluidity. Shortening the average acyl chain length lowers the temperature limit at which the transition from a liquid‐crystalline to a gel phase occurs. This adaptation helps to maintain membrane fluidity, which is essential for the survival and growth of bacteria (Russell, 2002). In this study, we observed that LCFAs are the predominant type of FAs for all the studied bacteria as was previously shown (Mező et al., 2022; Řezanka & Sigler, 2009). Earlier, it was reported that the production of MCFAs can naturally occur in both Gram‐negative and Gram‐positive bacteria (Ahn et al., 2023) and we detected the production of MCFAs in trace amounts in mainly Gram‐negative bacteria from genera *Pseudomonas*, *Shewanella*, *Acinetobacter* and *Flavobacterium*. Furthermore, we found that the production of MCFAs increased along with temperature increases for the genera *Pseudomonas*, *Acinetobacter* and *Flavobacterium*. It is known that both saturated and monounsaturated VLCFAs are present in almost all organisms but are predominantly found in very small quantities (Kyselová et al., 2022). We detected the presence of VLFAs in all bacteria and a few Gram‐positive bacteria, specifically those from the genera *Micrococcus* and all Firmicutes exhibited relatively high production of VLCFAs. In the case of *Rhodococcus*, it could originate from the mycolic acids layer present in the cell wall and similar results were previously reported for this genus (Nishiuchi et al., 2000). Overall, an increase in the production of VLCFAs with temperature decrease was observed for the majority of the studied bacteria. For some bacteria, the amount of VLCFAs increased from 5°C to 15°C and decreased from 15°C to 25°C.

Further, higher cultivation temperatures led to an increase in the amount of br‐SFAs in Gram‐positive bacteria and n‐SFAs in Gram‐negative bacteria, while lower temperatures led to an increase in the amount of n‐MUFAs in all Gram‐negative bacteria, and Gram‐positive *Rhodococcus* and *Carnobacterium*. In some cases, different genera from the same phylum showed distinct fatty acid profiles varying from the common Gram‐specific pattern. Thus, *Shewanella* strains contained br‐SFAs that were not detected for bacteria from other Proteobacteria genera but were previously shown in the literature (Skerratt et al., 2002). Furthermore, a significant effect of temperature on the FA profile of *Shewanella* was detected, indicating a high adaptability of this bacteria to environmental changes which was also pointed out by other researchers (Kloska et al., 2020; Skerratt et al., 2002; Wang et al., 2009). While n‐MUFAs were major FAs when *Shewanella* strains were grown at 5°C, br‐SFAs were predominant when the strains were grown at 15 and 25°C. The presence of br‐SFAs was also detected in *Flavobacterium* from the Bacteroidetes phylum. We also observed that Gram‐positive Actinobacteria from the genus *Rhodococcus* and Firmicutes from genera *Facklamia* and *Carnobacterium* had fatty acid profiles similar to Proteobacteria and were characterized by the predominance of n‐MUFA and n‐SFAs.

Besides the temperature‐triggered changes in the main fatty acids, we also observed temperature‐dependent production of some minor fatty acids. For example, hydroxy fatty acids (OH‐FA) detected in *Pseudomonas* and *Flavobacterium* and previously reported for these genera (Mező et al., 2022; Yano et al., 1976) showed an increase with the temperature decrease, which was in agreement with the previously reported results (LaliLingfa et al., n.d.; Kumar et al., 2002; Mező et al., 2022). The hydroxyl groups of these FAs are likely to serve a similar function as the branched fatty acids in phospholipid membranes, helping to maintain the membrane's viscous state at lower temperatures (LaliLingfa et al., n.d.; Kumar et al., 2002; Mező et al., 2022). A small amount of cyclic fatty acids (cyclic‐FA) produced in *Pseudomonas* increased with the increase of cultivation temperature which could be due to because cyclic fatty acids stabilize the membranes of bacteria by reducing the fluidity and improving their resistance to environmental stress. Production of cyclic fatty acids in small amounts has been found previously in Gram‐negative bacteria (Caligiani & Lolli, 2018). Some strains produced PUFAs, for example *Pseudomonas* sp. BIM B‐1674 produced up to 18% of PUFAs of the total fatty acid content, and *P. lundensis* BIM B‐1554 produced up to 10% of PUFAs when grown at 15°C and 25°C. Production of PUFA by Antarctic bacteria was previously reported (Jadhav et al., 2010; Nichols et al., 1993). Interestingly, we did not observe an increase in PUFA production at low temperatures as is often reported. And even for some Gram‐positive bacteria, we observed an opposite pattern of PUFA increase along with temperature increase. In this study, the production of PUFAs in *S. baltica*, a species previously positioned for PUFA production (Gentile et al., 2003) was not observed, which could be attributed to the preference of alternative mechanisms for maintaining membrane fluidity. The ratio between branched saturated fatty acids (br‐SFAs) and non‐methylene‐interrupted n‐MUFAs significantly decreased with decreasing temperature, indicating a different adaptation strategy for membrane stability as it was mentioned above.

In addition to traditional GC techniques, we utilized FTIR spectroscopy to expand knowledge on changes in lipids and other cellular components triggered by temperature. FTIR can provide information on the relative total lipid content, chain length and unsaturation of lipids, presence of different lipid classes, such as acyl glycerides, free fatty acids, polyesters, and it has been widely used for lipid analysis (Dean et al., 2010; Derenne et al., 2013; Forfang et al., 2017; Kosa, Kohler, et al., 2017; Kosa, Shapaval, et al., 2017; Kosa, Vuoristo, et al., 2018; Kosa, Zimmermann, et al., 2018; Shapaval et al., 2014; Shapaval et al., 2019). In addition, FTIR spectroscopy is an ideal tool for mapping the total cellular biochemical profile as it provides information on all main biomolecules: lipids, proteins, polyester, polysaccharides, phosphorus‐based compounds such as phospholipids, polyphosphates and so on (Alvarez‐Ordóñez et al., 2011; Kamnev, 2008).

FTIR analysis of the bacterial biomass obtained after cultivation at different temperatures showed some genera‐specific differences. For example, *Rhodococcus* bacteria had the highest intensity of peaks related to ‐C‐H (CH_2_) stretching and presence of peak =C‐H stretching of cis‐alkene HC=CH group found in polyunsaturated lipids that could be connected to the possible production of mycolic acids (Liu et al., 1996) or triacylglycerols (TAGs) (Alvarez et al., 2021) what was also detected by GC. Spectra of *Flavobacteria*, *Shewanella* and *Acinetobacter* showed an additional peak at 1728 cm^−1^ often associated with the presence of polyesters (Kamnev et al., 2021) and could indicate the production of PHAs (Christensen et al., 2023), and exploring these bacteria further would be important as bacterial polyesters are an important source of bioplastic.

FTIR analysis has shown that temperature fluctuations may induce considerable genera‐specific changes in protein structure for some bacteria, for example, *Shewanella*, *Pseudomonas* and *Carnobacterium*, where a shift to lower wavenumbers was detected for amide I peak at 1640 cm^−1^ related to β‐sheet structures of proteins. This might be associated with the decrease in the strength of the hydrogen bond of proteins due to the changes in protein conformation under temperature stress. For example, a decrease in hydrogen bond strength can be observed for the amide I band when proteins are denatured. However, it is important to note that shifts in the protein region can also be influenced by other factors, such as changes in protein–protein or protein‐ligand interactions (Barth & Zscherp, 2002).

The most significant temperature‐related alterations were recorded for the mixed region 1200–900 cm^−1^, where signals related to carbohydrates, nucleic acids, and phosphates are expected. Changes in this region were detected for all bacteria. The spectral region between 1200 and 900 cm^−1^ is rich in signals originating from various components, such as DNA, phospholipids, and complex sugar modes. Within this range, there are distinct and strong absorbance bands that have been observed in different bacteria and attributed to specific components of the cell wall (Kochan et al., 2018). As it was previously shown by (Kochan et al., 2018), phosphodiester groups (found in DNA, phospholipids, and teichoic acids/ lipoteichoic acid) create bands at around 1080 and 1220 cm^−1^ for symmetric and asymmetric PO2− stretching vibrations. Cell walls in Gram‐positive bacteria contain teichoic acids/ lipoteichoic acid as an additional phosphate compound compared to Gram‐negative cell walls. Phospholipids in the inner membrane of Gram‐positive bacteria may also contribute, but to a lesser extent. Gram‐negative bacteria have more phospholipids in their additional outer membrane. Overall, Gram‐positive bacteria have higher phosphodiester content, while Gram‐negative bacteria have more phospholipids.

Estimation of various ratio parameters using FTIR spectra showed that alterations in the L/P ratio were strictly genus‐specific and correlated well with the GC–FID results of the total lipid content which is an additional proof of the high sensitivity of FTIR spectroscopy for lipid analysis. These results indicate that temperature adaptation involves not only alterations in lipids but also modifications in protein structure, with minimal impact on protein concentration in cells. A significant effect of temperature on phospholipids and other phosphorus‐containing compounds was detected by calculating the 1083/1654 cm^−1^ ratio. For all studied bacteria except *Salinibacterium* and *Carnobacterium*, a significant increase in the ratio between phosphorus‐based compounds and proteins with temperature downshift was observed. The increased production of phosphorus‐containing compounds at low temperatures may have a connection to the increased synthesis of phospholipids, as it was shown by Gao et al. (2019). In that study, they observed an increase of total lipids and phospholipids in *Shewanella putrefaciens* along with a temperature decrease. On the other hand, they noticed a decrease in glycerolipids, sphingolipids, and saccharolipids at lower temperatures. This suggests a possible shift in lipid composition towards an increased proportion of phospholipids in response to lower temperatures. Also, an increase in absorbance for the peak responsible for phosphorus‐containing compounds at 1083 cm^−1^ for some bacteria at lower temperatures could have a connection to an increase in the total content of nucleic acids in bacterial cells that could be related to fluctuations in growth rate (Bates et al., 1985; Kochan et al., 2020).

A dataset obtained from measurements using a single technique can only provide insights from a single perspective. In this study, we employed a combination of analytical techniques—HTS‐FTIR, a rapid non‐destructive technique, and GC, a traditional analytical technique, to examine alterations in lipids and other biomolecules of Antarctic bacteria grown at different temperatures. The results from the correlation analysis show that for the majority of the studied bacteria, the correlation between the L/P ratio measured by FTIR spectroscopy and total lipid content measured by GC had moderate (*r* around 0.6) or strong (*r* around 0.8) linear relationships. The correlation within each genus varied after cultivation at different temperatures. However, for the majority of the genera, there is a higher correlation at moderate temperature (25°C) compared to 5°C and 15°C.

This study shows both the environmental and biotechnological importance of Antarctic bacteria. It has been observed that some Antarctic bacteria can accumulate lipids up to 20% at low temperatures. It might be interesting to explore further and investigate whether it is possible to establish lipid production by these bacteria. In addition, some bacteria were able to produce fatty acids of special industrial interest such as mycolic acid and branched unsaturated fatty acids.

## CONCLUSION

This study is one of the few previously published reporting comprehensive data on lipid and overall cellular biochemical profile and its temperature‐triggered changes for cold‐adapted bacteria. We showed that bacteria isolated from cold environments possess a taxonomy‐aligned fatty acid profile as it was earlier reported for bacteria from other environments. Our findings indicate that temperature variations may induce some modifications in cellular lipids. These alterations encompass changes in the total lipid content, fatty acid composition, and lipid classes. Additionally, we observed notable transformations in other cellular components such as proteins and phosphorus‐containing compounds. These changes are taxonomy‐specific, meaning that despite of principle similarity in cell structure bacteria do not have a single common adaptation mechanism to temperature fluctuations and often show different chemical responses.

## AUTHOR CONTRIBUTIONS

**Volha Akulava:** Conceptualization (equal); formal analysis (equal); investigation (equal); methodology (equal); data curation (equal); validation (equal); visualization; writing – original draft (equal); writing – review and editing (equal). **Margarita Smirnova:** Conceptualization (equal); investigation (equal); writing – original draft (equal); writing – review and editing (equal). **Dana Byrtusova:** Investigation (equal); writing – review and editing (equal). **Boris Zimmermann:** Data curation (equal); methodology (equal); writing – review and editing (equal). **Dag Ekeberg:** Data curation (equal); investigation (equal); writing – review and editing (equal). **Achim Kohler:** Funding acquisition; data curation (equal); supervision; writing – review and editing (equal). **Uladzislau Blazhko:** Formal analysis (equal); validation (equal); writing – review and editing (equal). **Uladzislau Miamin:** Conceptualization (equal);supervision;   writing – review and editing (equal). **Leonid Valentovich:** Conceptualization (equal); supervision; writing – review and editing (equal). **Volha Shapaval:** Conceptualization (equal); funding acquisition; validation (equal); supervision;  writing – review and editing (equal).

## CONFLICT OF INTEREST STATEMENT

The authors declare no conflicts of interest.

## Supporting information


**DATA S1.** Supporting Information.

## Data Availability

All data sets generated for this study are available in the Zenodo repository: https://zenodo.org/doi/10.5281/zenodo.10051607
