# Supplementary material for: Explorative characterization and taxonomy‐aligned comparison of alterations in lipids and other biomolecules in Antarctic bacteria grown at different temperatures
Source: Environ Microbiol Rep. 2024 Feb 3;16(1):e13232. doi: 10.1111/1758-2229.13232 (PMC10878007; doi:10.1111/1758-2229.13232)
Supplement: Supplementary file 1 — DATA S1. Supporting Information. [file EMI4-16-e13232-s001.docx]

**Supplementary materials**

Table S1. Gen Bank accession numbers of bacteria used in this study

| Phylum | Genus | Strain name and collection № | Gen Bank Accession Number |
| --- | --- | --- | --- |
| Proteobacteria | *Polaromonas* | *Polaromonas sp*. BIM B–1676 ^GS^** | MT890199 |
|  | *Pseudomonas* | *Pseudomonas extremaustralis* BIM B–1672 ^GS^ | MT890192 |
|  |  | *Pseudomonas fluorescens* BIM B–1668 ^GS^ | MT89019 |
|  |  | *Pseudomonas leptonychotis* BIM B–1559^MP^ | ON248066 |
|  |  | *Pseudomonas leptonychotis* BIM B–1568^MP^ | ON248076 |
|  |  | *Pseudomonas leptonychotis* BIM B–1566^MP^ | ON248077 |
|  |  | *Pseudomonas lundensis* BIM B–1554^MP^ | ON248061 |
|  |  | *Pseudomonas lundensis* BIM B–1555^MP^ | ON248062 |
|  |  | *Pseudomonas lundensis* BIM B–1556^MP^ | ON248063 |
|  |  | *Pseudomonas peli* BIM B–1560^MP^ | ON248067 |
|  |  | *Pseudomonas peli* BIM B–1569^MP^ | ON248075 |
|  |  | *Pseudomonas peli* BIM B–1546 ^MP^ | ON248078 |
|  |  | *Pseudomonas peli* BIM B–1552^MP^ | ON248080 |
|  |  | *Pseudomonas peli* BIM B–1542^MP^ | ON248083 |
|  |  | *Pseudomonas peli* BIM B–1548^MP^ | ON248084 |
|  |  | *Pseudomonas sp*. BIM B–1635^GS^ | MT890174 |
|  |  | *Pseudomonas sp.* BIM B–1667^GS^ | MT890189 |
|  |  | *Pseudomonas sp*. BIM B–1673^GS^ | MT890191 |
|  |  | *Pseudomonas sp*. BIM B–1674^GS^ | MT890193 |
|  | *Psychrobacter* | *Psychrobacter glacinicola* BIM B–1629^GS^** | MT890168 |
|  |  | *Psychrobacter urativorans* BIM B–1655 ^GS^** | MT890181 |
|  |  | *Psychrobacter urativorans* BIM B–1662^GS^** | MT890182 |
|  | *Shewanella* | *Shewanella baltica* BIM B–1565^MP^ | ON248060 |
|  |  | *Shewanella baltica* BIM B–1557^MP^ | ON248064 |
|  |  | *Shewanella baltica* BIM B–1561^MP^ | ON248069 |
|  |  | *Shewanella baltica* BIM B–1563^MP^ | ON248072 |
|  | *Acinetobacter* | *Acinetobacter lwoffii* BIM B–1558^MP^ | ON248065 |
| Bacteroidetes | *Flavobacterium* | *Flavobacterium degerlachei* BIM B–1562^MP^ | ON248071 |
| Actinobacteria | Agrococcus | *Agrococcus citreus* BIM B–1547^MP^ | ON248081 |
|  | *Arthrobacter* | *Arthrobacter agilis* BIM B–1543 ^MP^ | ON248082 |
|  |  | *Arthrobacter cryoconiti* BIM B–1627^GS^** | MT890166 |
|  |  | *Arthrobacter oryzae* BIM B–1663^GS^ | MT890183 |
|  |  | *Arthrobacter sp*. BIM B–1624^GS^** | MT890163 |
|  |  | *Arthrobacter sp*. BIM B–1625 ^GS^ | MT890164 |
|  |  | *Arthrobacter sp*. BIM B–1626^GS^** | MT890165 |
|  |  | *Arthrobacter sp*. BIM B–1628^GS^** | MT890167 |
|  |  | *Arthrobacter sp*. BIM B–1664^GS^ | MT890186 |
|  |  | *Arthrobacter sp*. BIM B–1666^GS^** | MT890188 |
|  |  | *Arthrobacter sp*. BIM B–1656^GS^ | MT890194 |
|  |  | *Arthrobacter sp*. BIM B–1549^MP^ | ON248073 |
|  | *Brachybacterium* | *Brachybacterium paraconglomeratum* BIM B–1571 ^MP^ | ON248074 |
|  | *Cryobacterium* | *Cryobacterium arcticum* BIM B–1619 ^GS^** | MT890158 |
|  |  | *Cryobacterium soli* BIM B–1620 ^GS^** | MT890159 |
|  |  | *Cryobacterium soli* BIM B–1658^GS^ | MT890196 |
|  |  | *Cryobacterium soli* BIM B–1659^GS^ | MT890197 |
|  |  | *Cryobacterium soli* BIM B–1677^GS^ | MT890198 |
|  |  | *Cryobacterium soli* BIM B–1675^GS^ | MT890200 |
|  | *Leifsonia* | *Leifsonia antarctica* BIM B–1631^GS^** | MT890170 |
|  |  | *Leifsonia antarctica* BIM B–1632^GS^ | MT890171 |
|  |  | *Leifsonia antarctica* BIM B–1637 ^GS^ | MT890176 |
|  |  | *Leifsonia antarctica* BIM B–1638 ^GS^ | MT890177 |
|  |  | *Leifsonia antarctica* BIM B–1639 ^GS^ | MT890178 |
|  |  | *Leifsonia antarctica* BIM B–1669 ^GS^ | MT890179 |
|  |  | *Leifsonia antarctica*. BIM B–1671^GS^ | MT890184 |
|  |  | *Leifsonia kafniensis* BIM B–1633^GS^ | MT890172 |
|  |  | *Leifsonia rubra* BIM B–1622 ^GS^** | MT890161 |
|  |  | *Leifsonia rubra* BIM B–1623 ^GS^ | MT890162 |
|  |  | *Leifsonia rubra* BIM B–1634 ^GS^** | MT890173 |
|  |  | *Leifsonia rubra* BIM B–1567^MP^ | ON248088 |
|  | *Micrococcus* | *Micrococcus luteus* BIM B–1545^MP^ | ON248079 |
|  | *Paeniglutamicibacter* | *Paeniglutamicibacter antarcticus* BIM B–1657^GS^** | MT890195 |
|  | *Rhodococcus* | *Rhodococcus erythropolis* BIM B–1660^GS^ | MT890201 |
|  |  | *Rhodococcus erythropolis* BIM B–1661^GS^ | MT890202 |
|  |  | *Rhodococcus yunnanensis* BIM B–1621^GS^** | MT890160 |
|  |  | *Rhodococcus yunnanensis* BIM B–1670^GS^ | MT890185 |
|  | *Salinibacterium* | *Salinibacterium sp*. BIM B–1630 ^GS^ | MT890169 |
|  |  | *Salinibacterium sp*. BIM B–1636^GS^ | MT890175 |
|  |  | *Salinibacterium sp*. BIM B–1654^GS^ | MT890180 |
|  |  | *Salinibacterium sp*. BIM B–1665^GS^ | MT890187 |
| Firmicutes | *Facklamia* | *Facklamia tabacinasalis* BIM B–1577^MP^ | ON248087 |
|  | *Sporosarcina* | *Sporosarcina sp*. BIM B–1539 ^MP^ | ON248068 |
|  | *Carnobacterium* | *Carnobacterium funditum* BIM B–1541^MP^** | ON248085 |
|  |  | *Carnobacterium iners* BIM B–1544^MP^** | ON248086 |
|  |  | *Carnobacterium inhibens* BIM B–1540^MP^ | ON248070 |


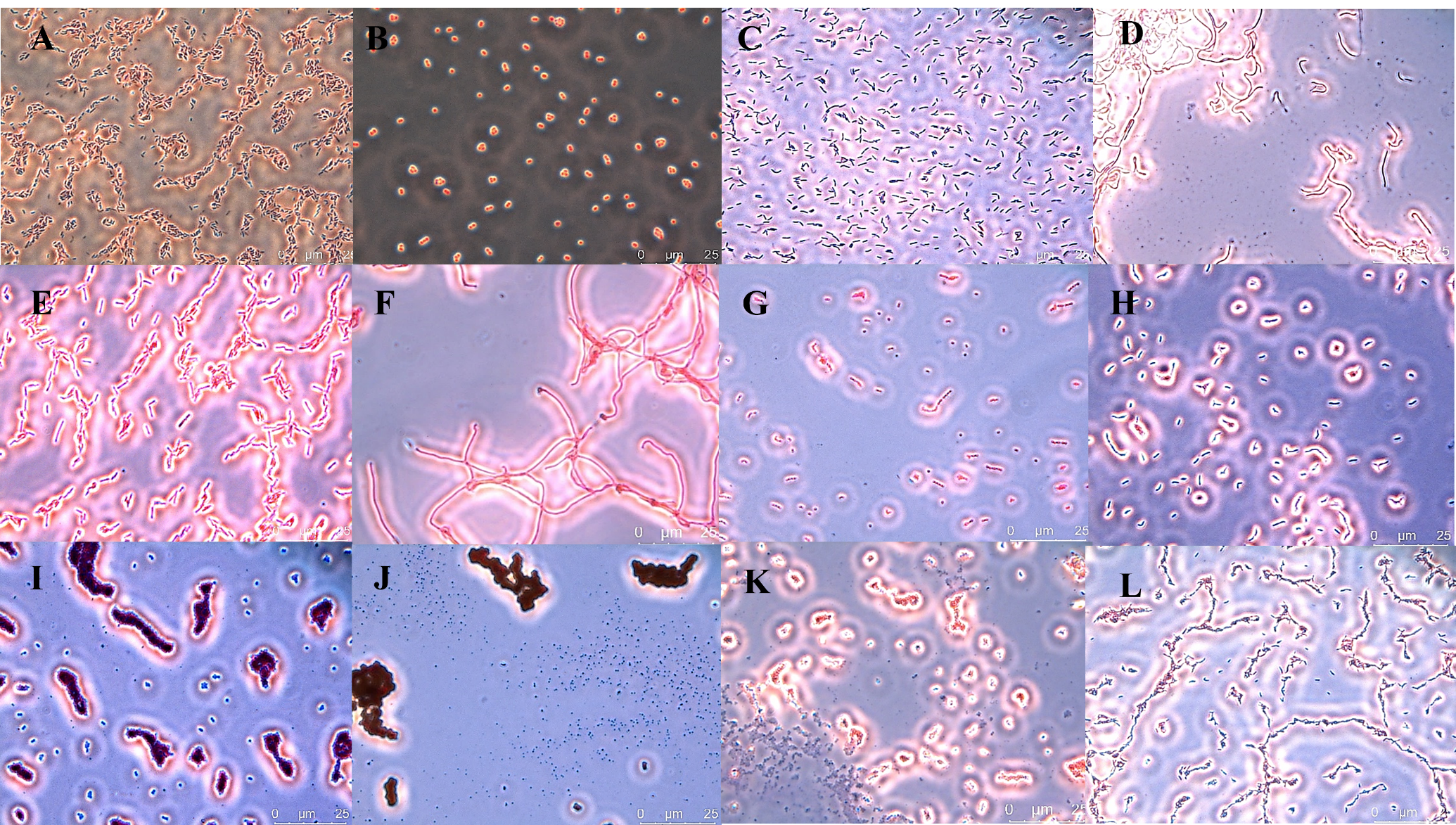


Figure S1– Gram-stained fast-growing bacteria isolated from the temporary meltwater ponds of the coastal area of East Antarctica: A - *Shewanella baltica* BIM B – 1563; B - *Acinetobacter lwoffii* BIM B – 1558; C - *Pseudomonas peli* BIM B – 1560; D - *Flavobacterium degerlachei* BIM B – 1562; E - *Sporosarcina* sp. BIM B – 1539; F - *Carnobacterium funditum* BIM B – 1541; G - *Facklamia tabacinasalis* BIM B – 1577; H - *Arthrobacter* sp. BIM B – 1549; I - *Brachybacterium paraconglomeratum* BIM B – 1571; J - *Micrococcus luteus* BIM B – 1545; K - *Agrococcus citreus* BIM B – 1547; L - *Leifsonia rubra* BIM B – 1567.

**
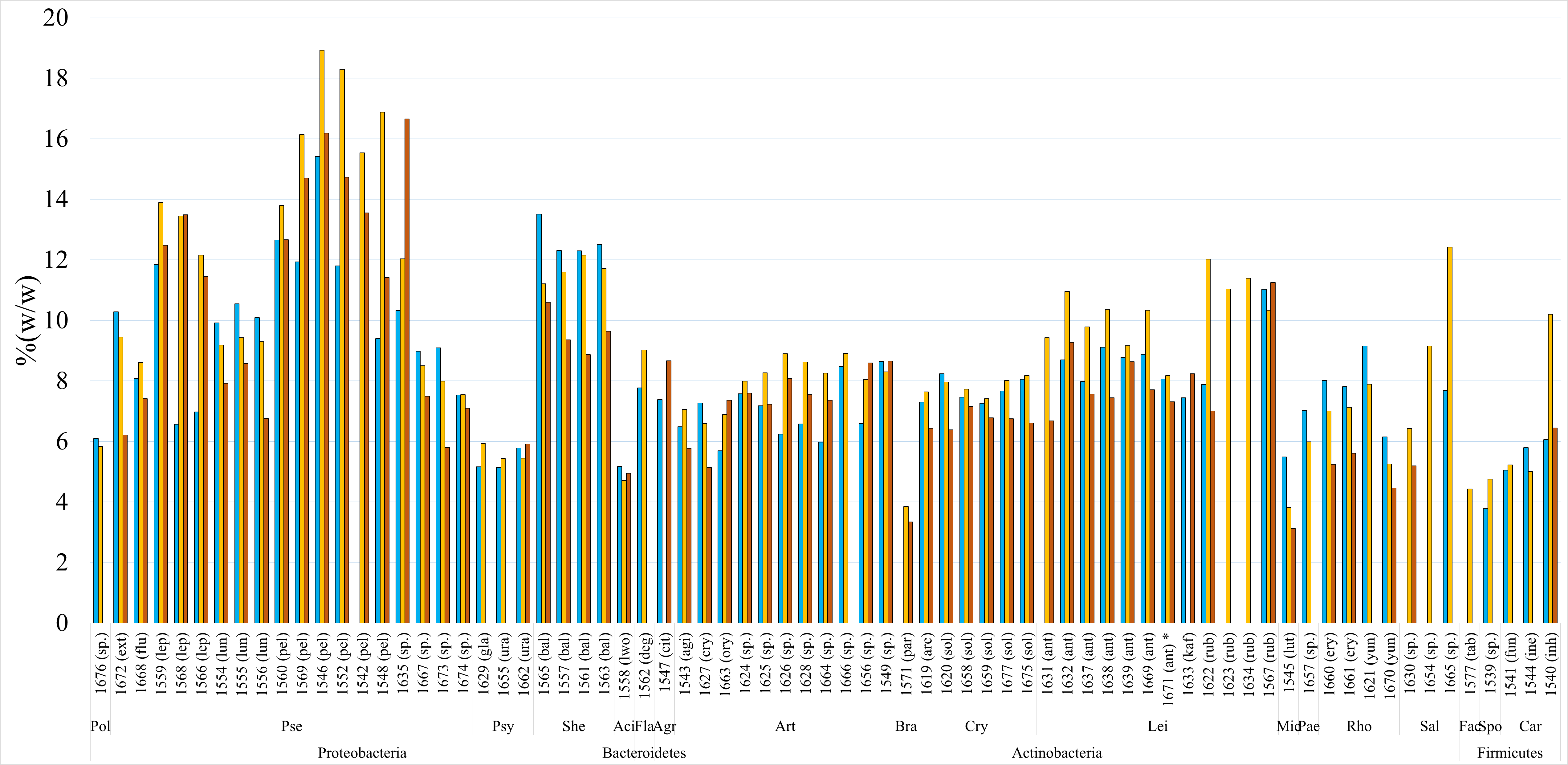
**

Figure S2. Total lipid content (%, w/w) of bacterial biomass grown at different temperatures (blue – 5°C, yellow – 15°C, and orange – 25°C).

**
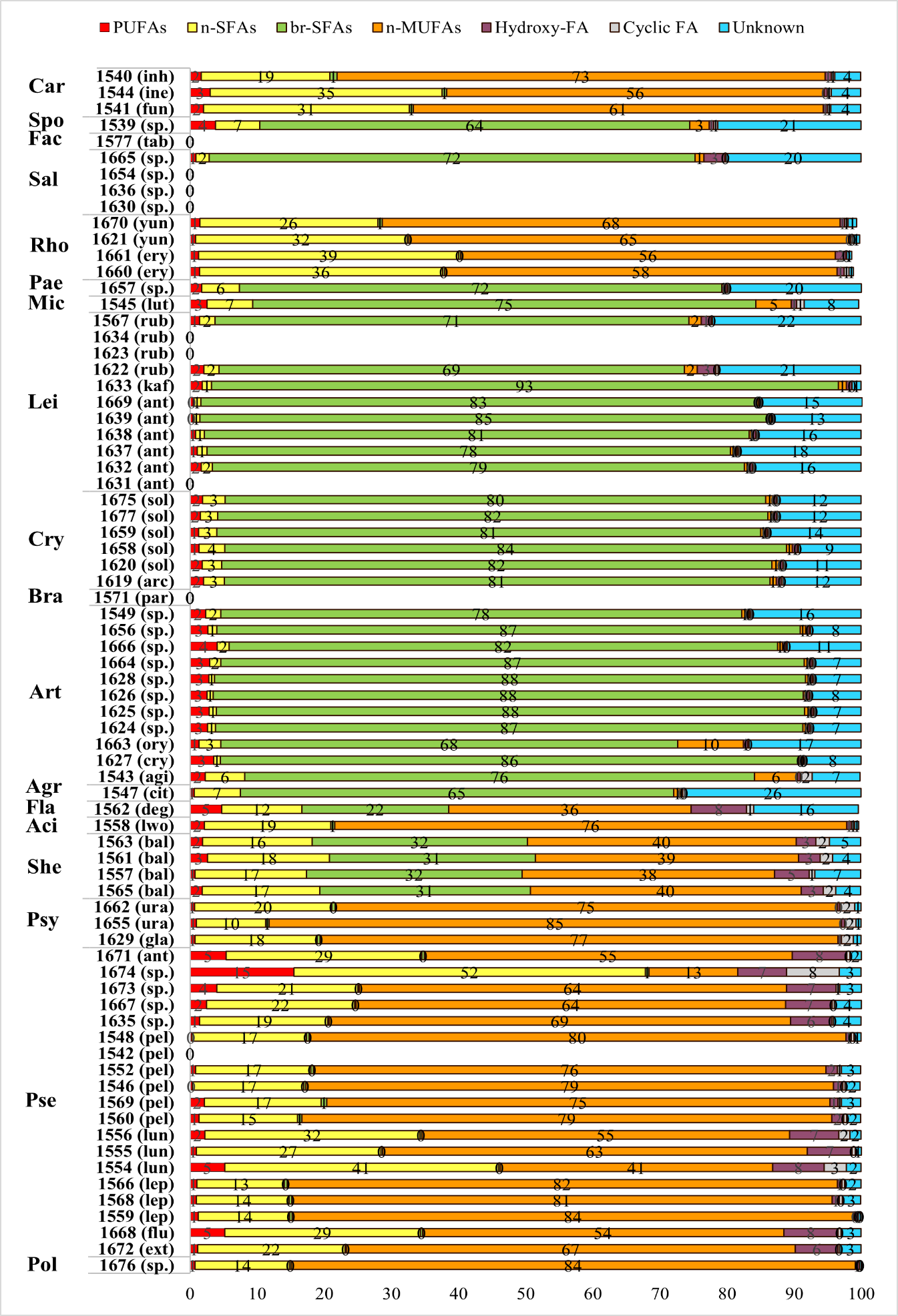
**

Figure S3. Fatty acid profile of Antarctic bacteria grown at 5°C (%, w/w).

**
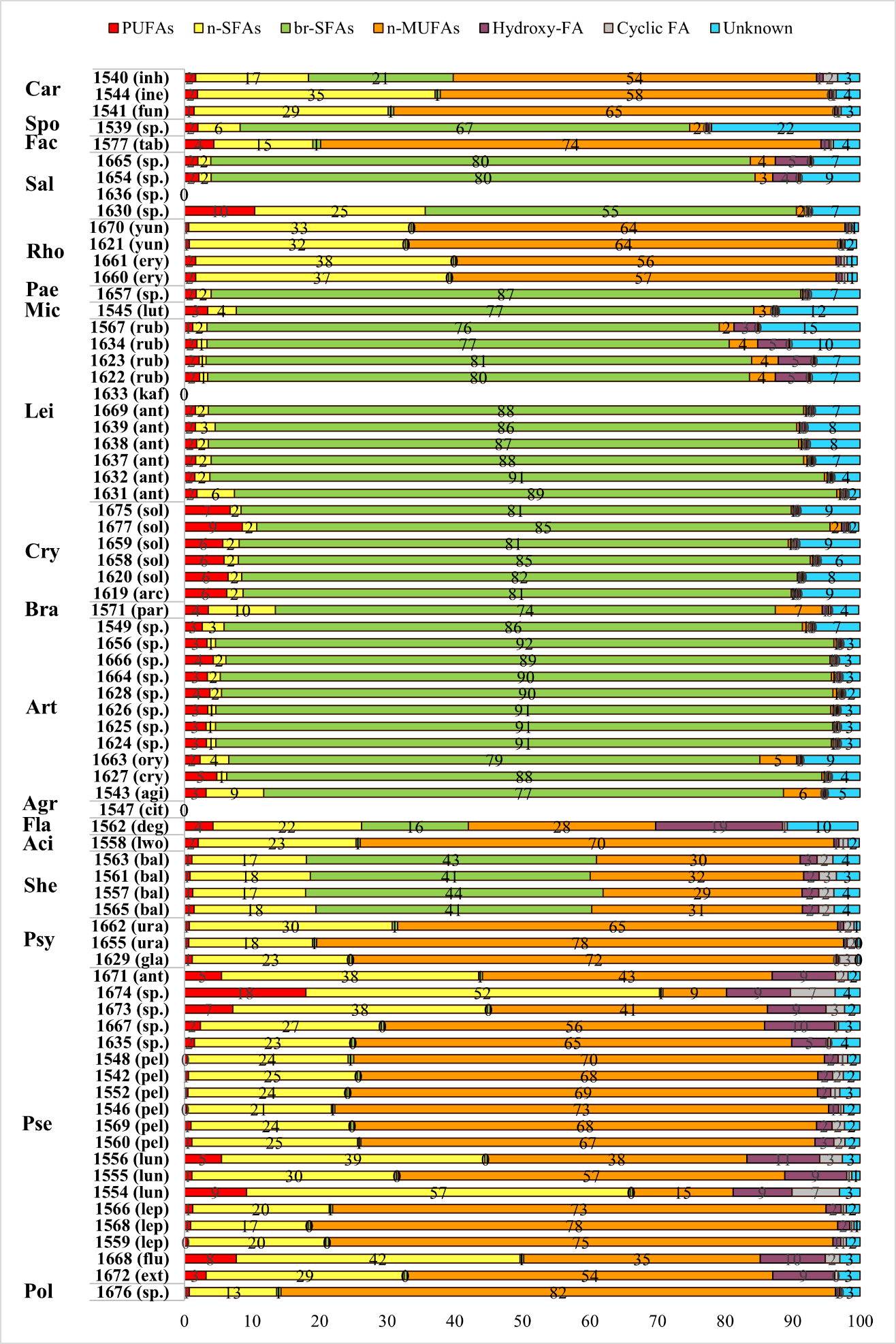
**

Figure S4. Fatty acid profile of Antarctic bacteria grown at 15°C (%, w/w).

**
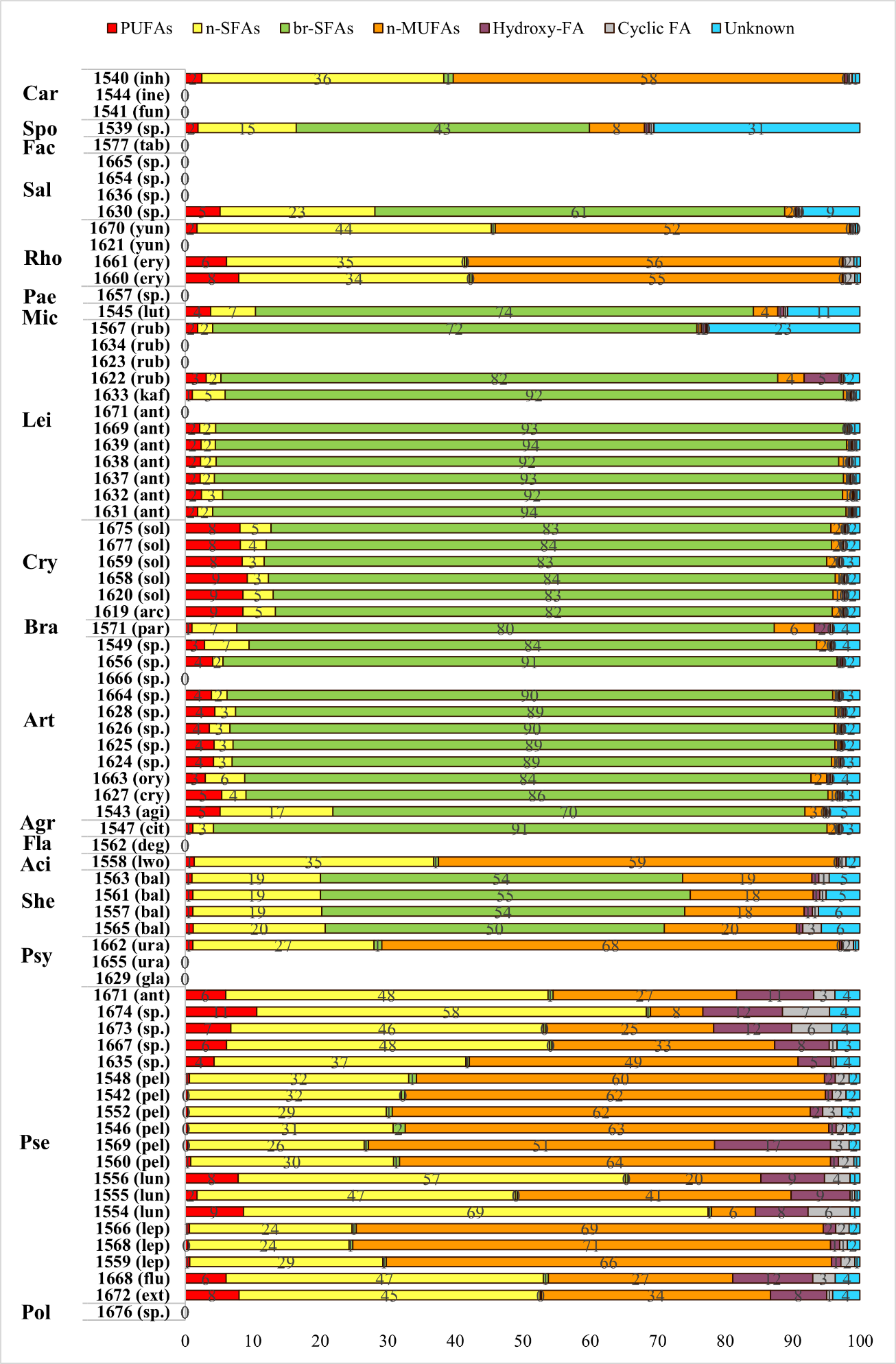
**

Figure S5. Fatty acid profile of Antarctic bacteria grown at 25°C (%, w/w).


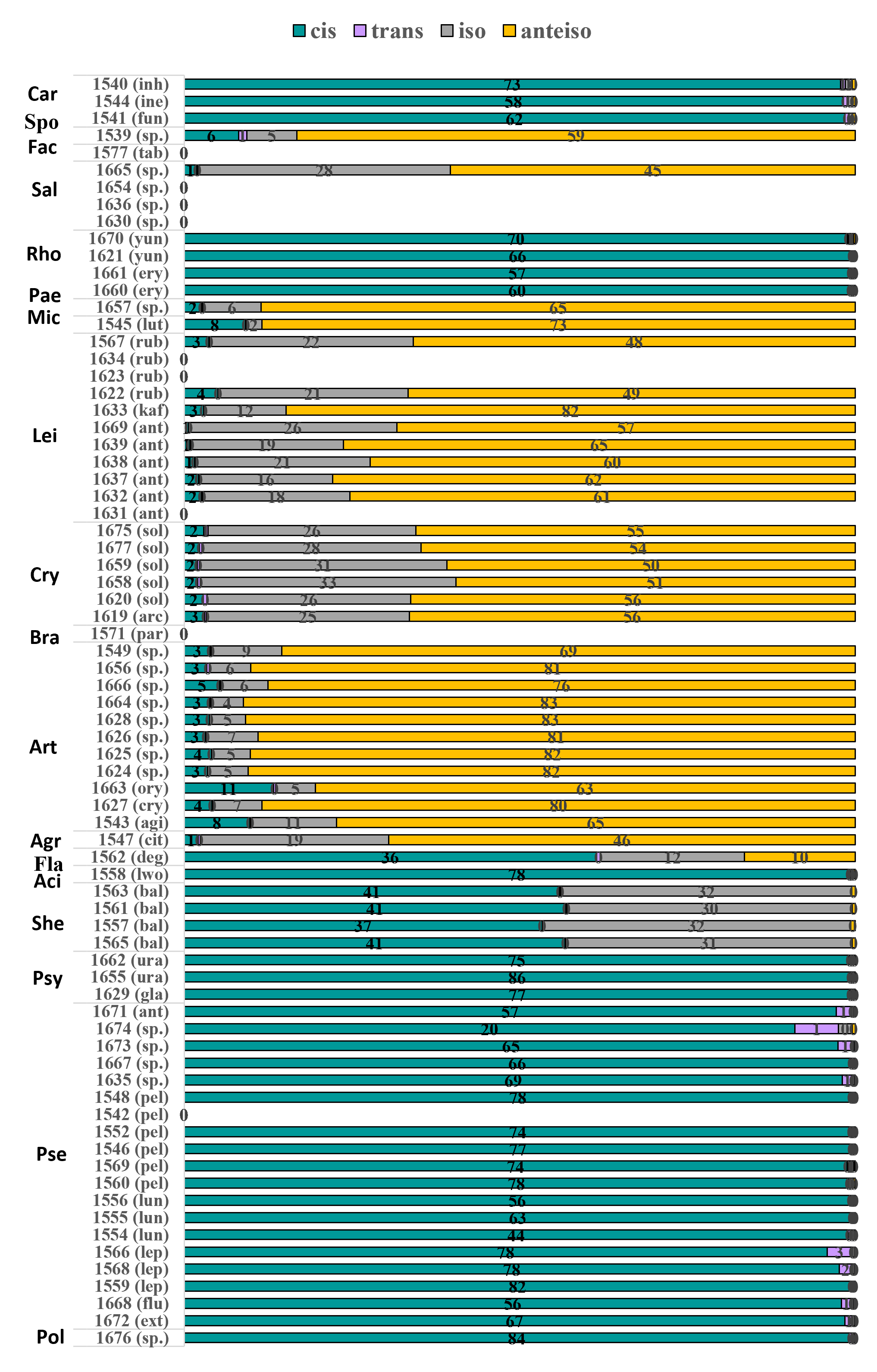


Figure S6. Fatty acid profile of Antarctic bacteria grown at 5°C (%, w/w).


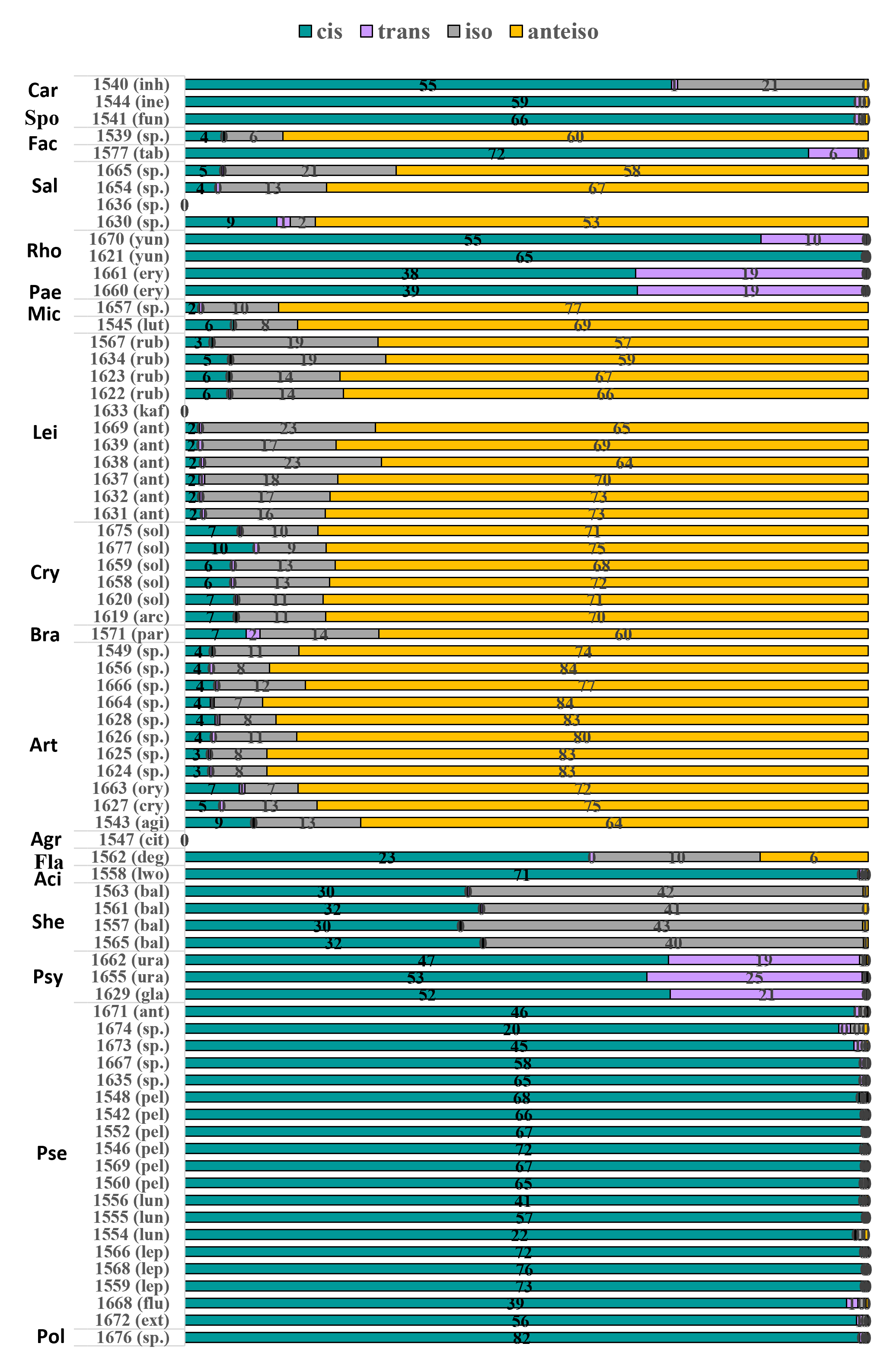


Figure S7. Fatty acid profile of Antarctic bacteria grown at 15°C (%, w/w).


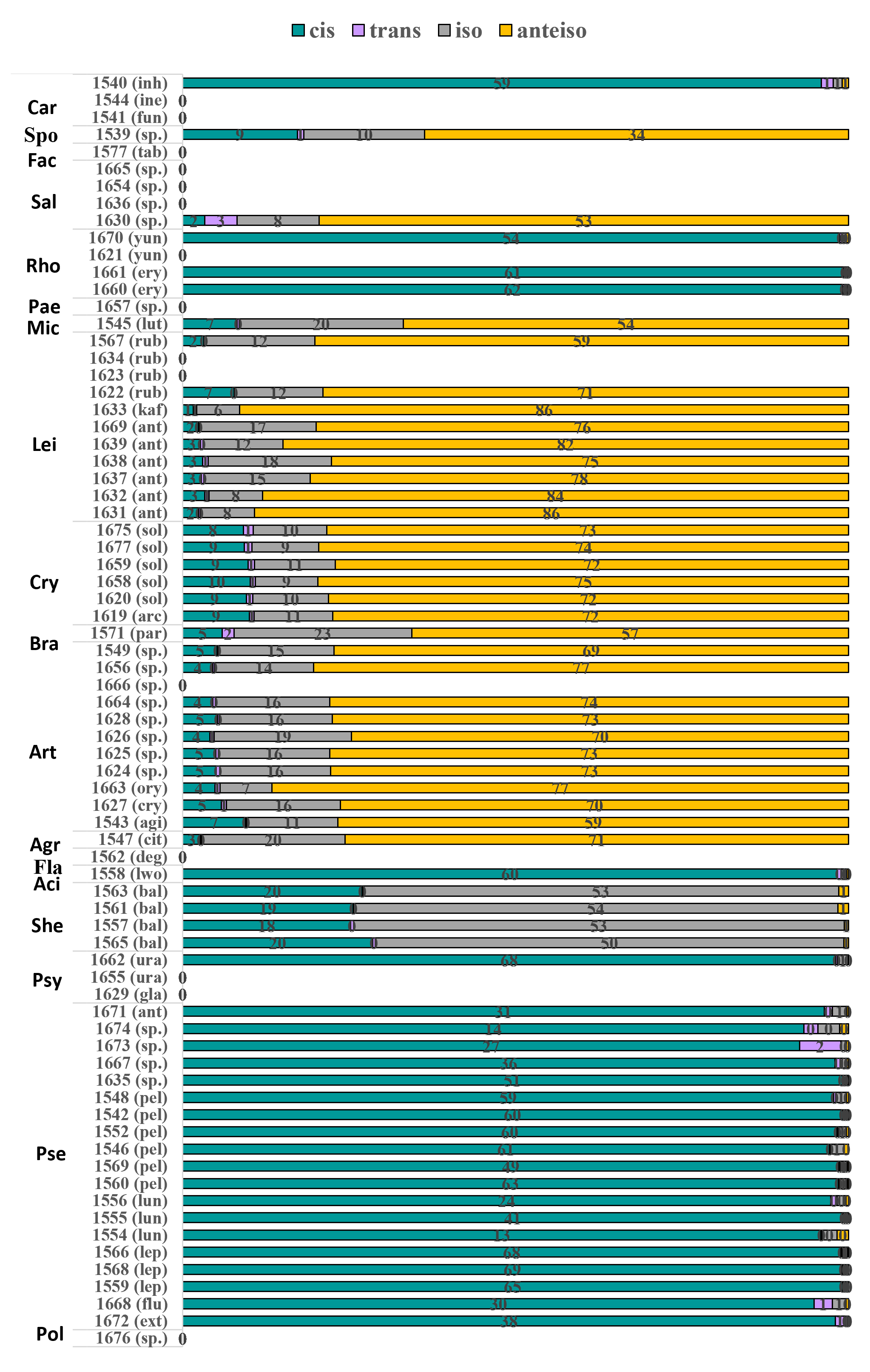


Figure S8. Fatty acid profile of Antarctic bacteria grown at 15°C (%, w/w).


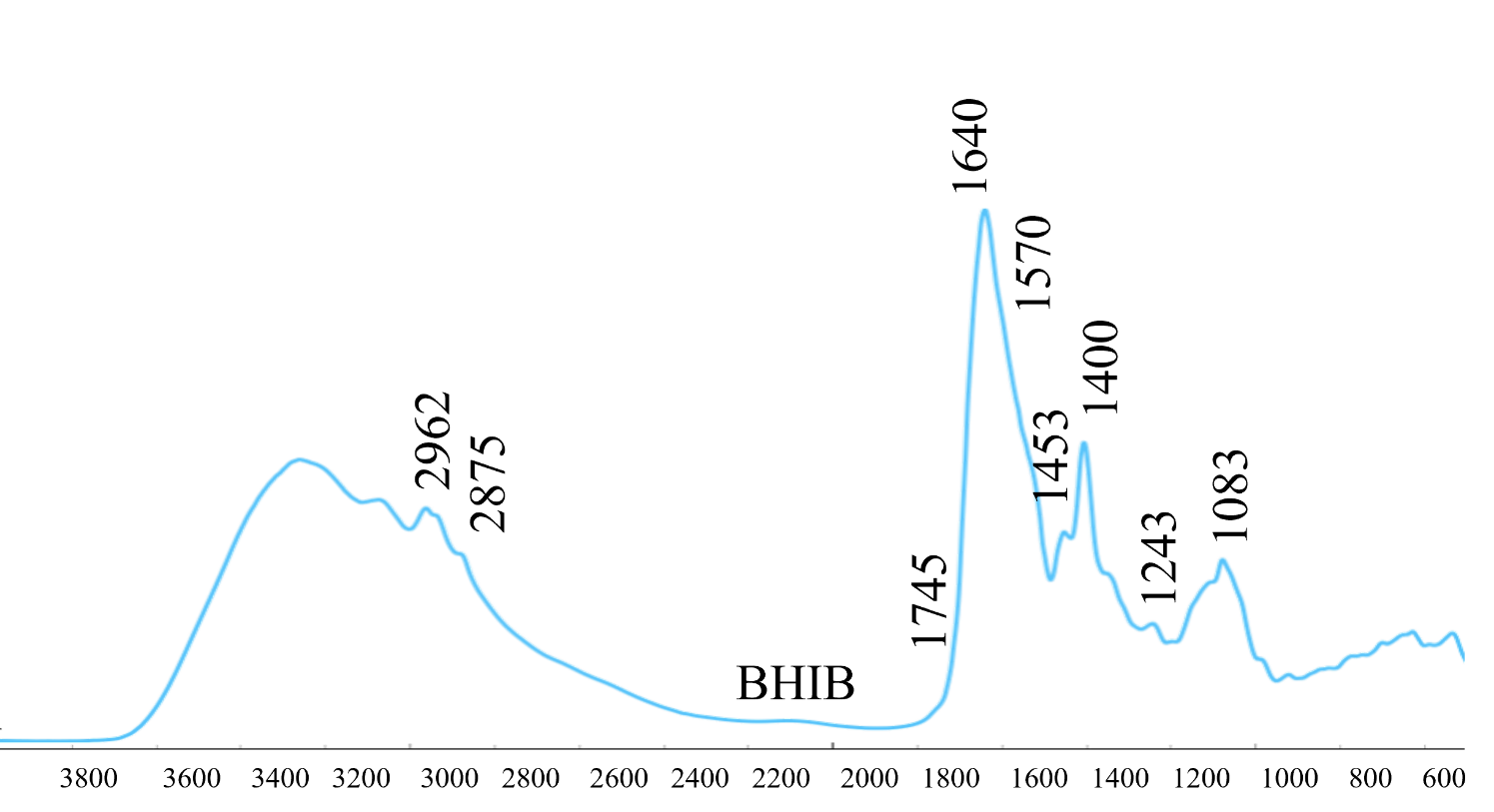


Figure S9. FTIR-HTS spectra of BHIB media.


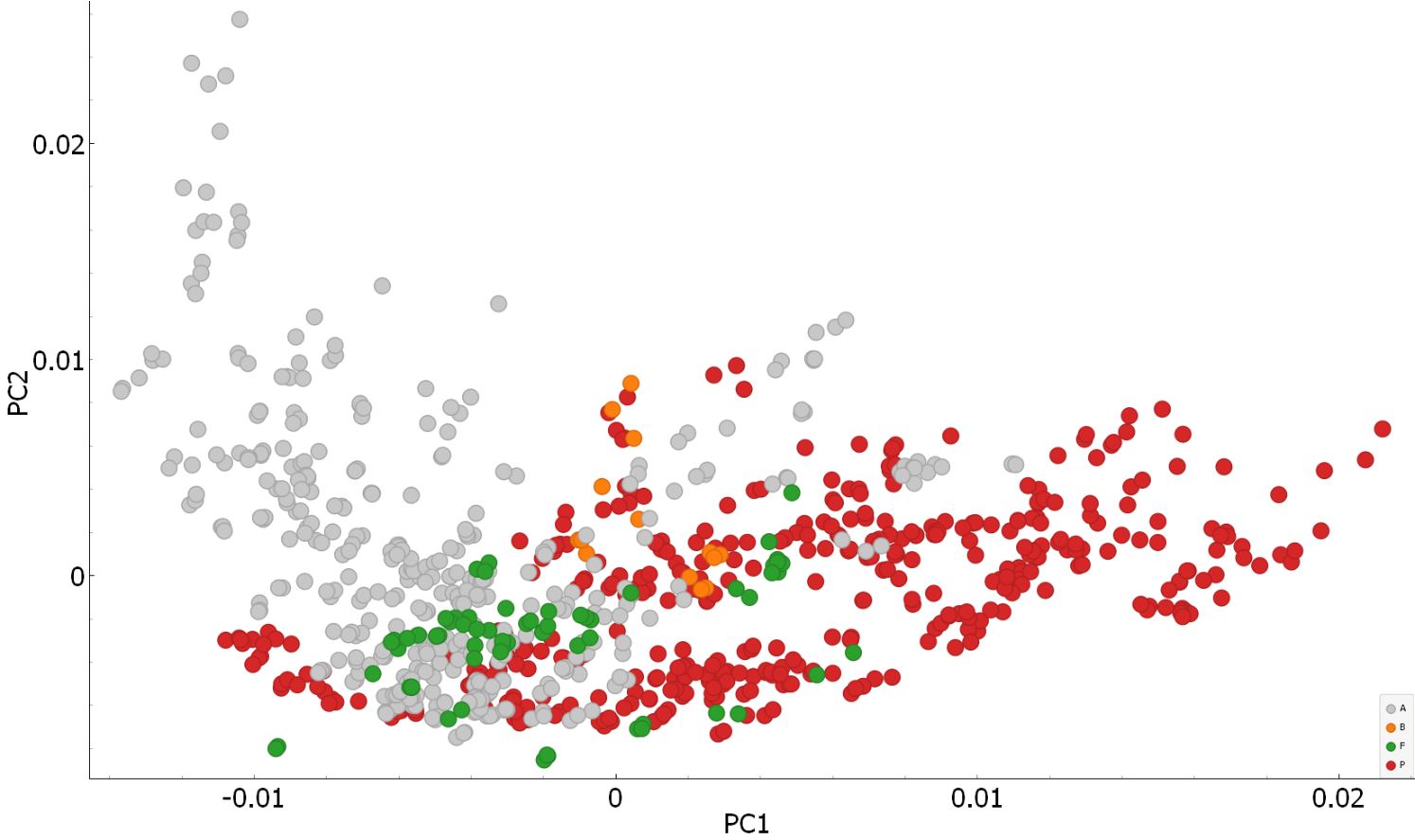


Figure S10. PCA plot on phyla level for the whole spectral region. P-Proteobacteria, A-Actinobacteria, F-Firmicutes, and B-Bacteroidetes.


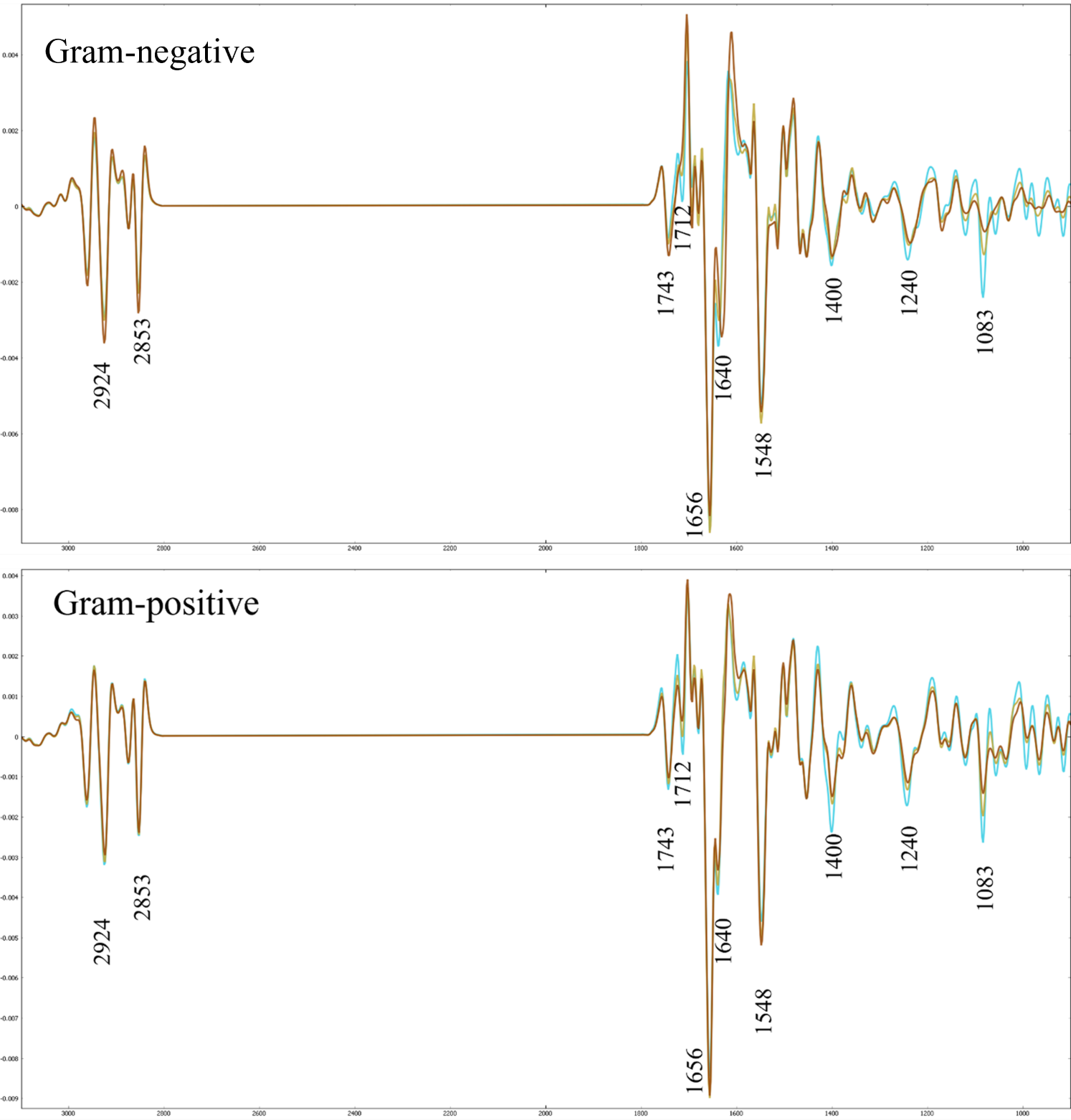


Figure S11. Second derivative FTIR spectra of bacterial biomass of different Gram groups averaged for different temperatures (blue – 5°C, yellow – 15°C, and orange – 25°C).


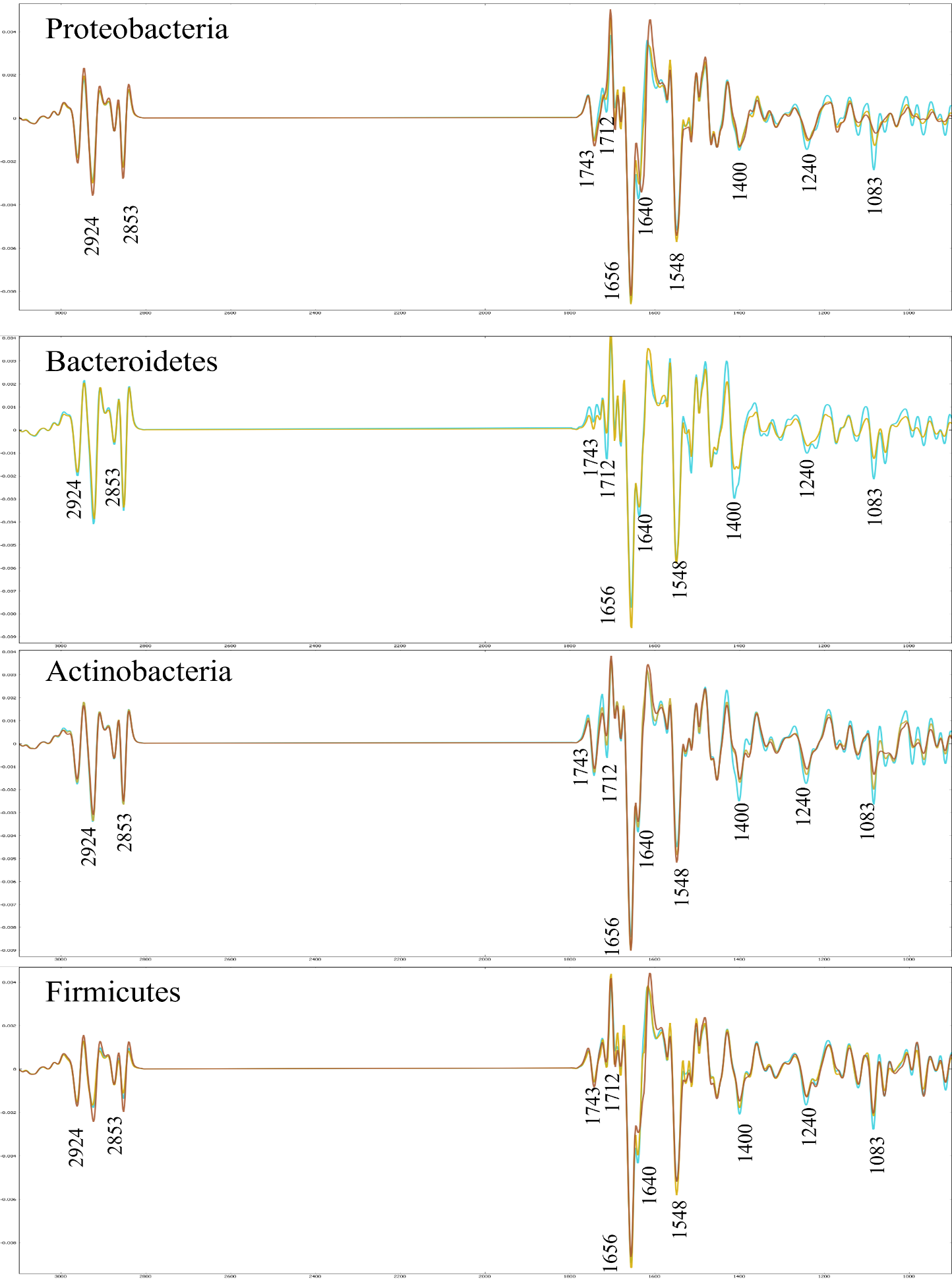


Figure S12. Second derivative FTIR spectra of bacterial biomass of different phyla averaged for different temperatures (blue – 5°C, yellow – 15°C, and orange – 25°C).


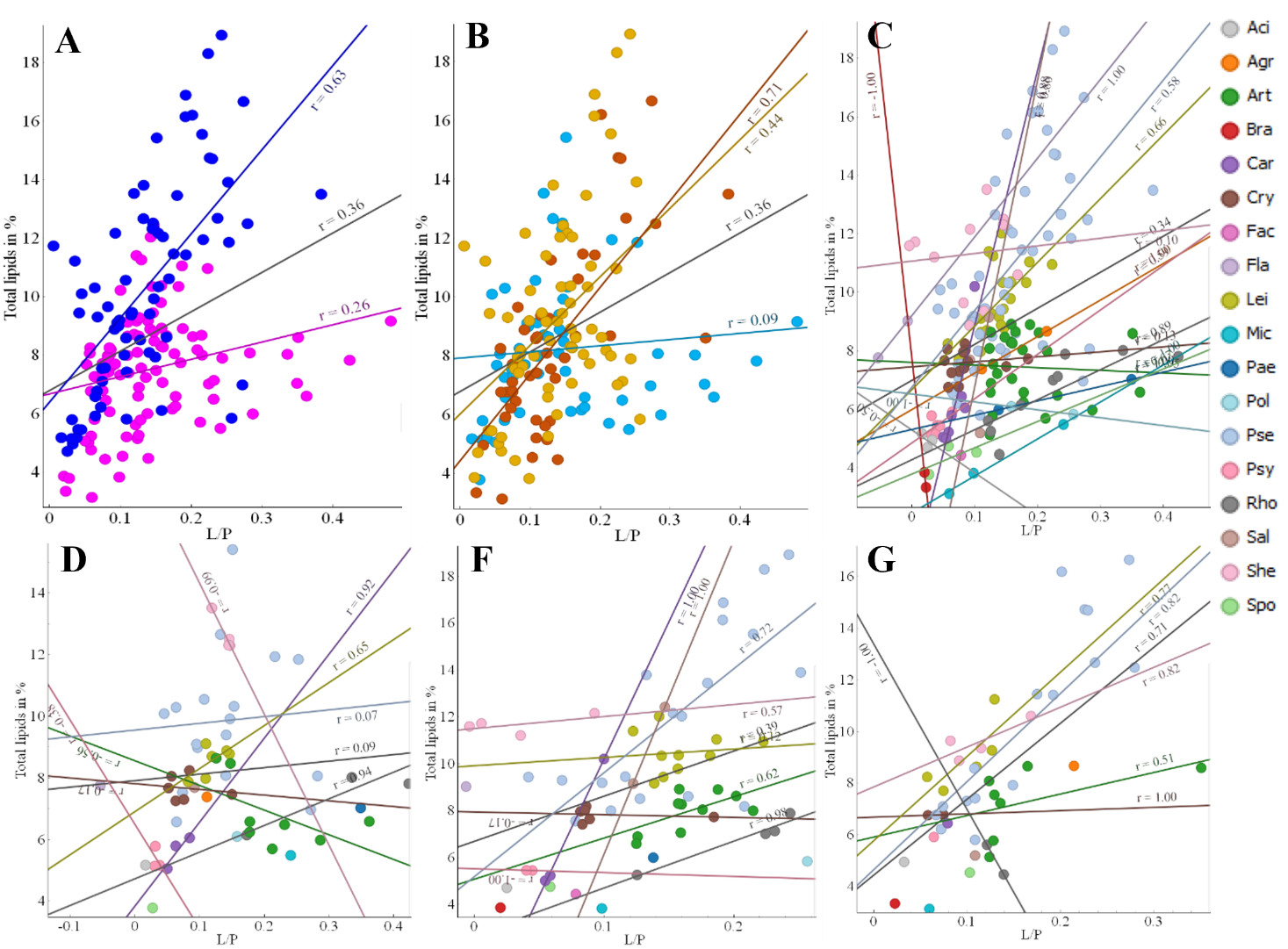


Figure S13. Pearson’s correlation coefficients between total lipids in % measured by GC and lipid to protein ratio calculated based on FTIR spectra: A – Gram-negative (blue) and gram-positive (pink) bacteria, B – different temperatures (blue – 5°C, yellow – 15°C and orange – 25°C), C – different genera, D – different genera cultivated at 5°C, F – different genera cultivated at 15°C, G – different genera cultivated at 25°C.

Figure S14. Averaged total lipid content (%, w/w) of bacterial biomass grown at different temperatures for Gram-negative and Gram-positive bacteria.
